# Organo‐Pt^ii^ Complexes for Potent Photodynamic Inactivation of Multi‐Drug Resistant Bacteria and the Influence of Configuration

**DOI:** 10.1002/advs.202306936

**Published:** 2024-01-31

**Authors:** Hui Chong, Xuanwei Liu, Siyu Fang, Xiaofei Yang, Yuefei Zhang, Tianyi Wang, Lin Liu, Yinshi Kan, Yueqi Zhao, Hongying Fan, Jingqi Zhang, Xiaoyu Wang, Hang Yao, Yi Yang, Yijian Gao, Qi Zhao, Shengliang Li, Martin Plymoth, Juqun Xi, Yu Zhang, Chengyin Wang, Huan Pang

**Affiliations:** ^1^ Department of Chemical and Chemical Engineering Yangzhou University No. 180, Si‐Wang‐Ting Rd. Yangzhou Jiangsu 225009 P. R. China; ^2^ Department of Emergency Affiliated Hospital of Yangzhou University Yangzhou Jiangsu 225000 China; ^3^ School of Nursing Yangzhou University Yangzhou 225009 P. R. China; ^4^ Jiangsu Key Laboratory of Integrated Traditional Chinese and Western Medicine for Prevention and Treatment of Senile Diseases No. 88 South University Rd. Yangzhou 225009 P. R. China; ^5^ Testing Center of Yangzhou University No. 48 Wenhui East Rd. Yangzhou 225009 P. R. China; ^6^ School of Materials Science and Engineering University of Science and Technology Beijing Beijing 100083 P. R. China; ^7^ Center Laboratory Affiliated Hospital of Yangzhou University Yangzhou 225009 P. R. China; ^8^ College of Pharmaceutical Sciences Soochow University Suzhou 215123 P. R. China; ^9^ Westmead hospital Sydney NSW 2145 Australia; ^10^ Department of Clinical Microbiology Umeå University Umeå 90187 Sweden; ^11^ Department of Pharmacology Institute of Translational Medicine School of Medicine Yangzhou University Yangzhou 225009 P. R. China; ^12^ Jiangsu Key Laboratory of Integrated Traditional Chinese and Western Medicine for Prevention and Treatment of Senile Diseases Yangzhou 225009 P. R. China; ^13^ Department of Chemical and Chemical Engineering Yangzhou University No. 180, Si‐Wang‐Ting Rd. Yangzhou Jiangsu 225009 P. R. China

**Keywords:** antibacterial agents, drug resistance, Pt^II^, photosensitizers, structure‐activity relationship, reactive oxygen species, membrane anchoring

## Abstract

Pt^II^ based organometallic photosensitizers (PSs) have emerged as novel potent photodynamic inactivation (PDI) reagents through their enhanced intersystem crossing (ISC) processes. Currently, few Pt^II^ PSs have been investigated as antibacterial materials, with relatively poor performances reported and with structure‐activity relationships not well described. Herein, a pair of configurational isomers are reported of Bis‐BODIPY (4,4‐difluoro‐boradizaindacene) embedded Pt^II^ PSs. The *cis*‐isomer (*cis*‐BBP) displayed enhanced ^1^O_2_ generation and better bacterial membrane anchoring capability as compared to the trans‐isomer (trans‐BBP). The effective PDI concentrations (efficiency > 99.9%) for cis‐BBP in Acinetobacter baumannii (multi‐drug resistant (MDR)) and Staphylococcus aureus are 400 nM (12 J cm^−2^) and 100 nM (18 J cm^−2^), respectively; corresponding concentrations and light doses for *trans*‐BBP in the two bacteria are 2.50 µM (30 J cm^−2^) and 1.50 µM (18 J cm^−2^), respectively. The 50% and 90% minimum inhibitory concentration (MIC_50_ and MIC_90_) ratio of trans‐BBP to *cis*‐BBP is 22.22 and 24.02 in *A. baumannii* (MDR); 21.29 and 22.36 in methicillin resistant *S. aureus* (MRSA), respectively. Furthermore, *cis*‐BBP displays superior in vivo antibacterial performance, with acceptable dark and photoinduced cytotoxicity. These results demonstrate *cis*‐BBP is a robust light‐assisted antibacterial reagent at sub‐micromolecular concentrations. More importantly, configuration of Pt^II^ PSs should be an important issue to be considered in further PDI reagents design.

## Graphical abstract

Herein, the first photoactive organo‐Pt^II^ configurational isomers (*cis*‐BBP and *trans*‐BBP) as potent in vitro and in vivo antibacterial reagents are reported. *Cis*‐BBP (effectively antibacterial at sub‐micromolecular concentrations) displays more powerful antibacterial efficiency comparing to *trans*‐BBP. The better ROS generating and membrane anchoring capability contributes to the enhanced antibacterial efficacy. The configuration of Pt^II^ is a significant issue in related photosensitizer design.

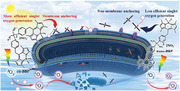

## 1 Introduction

Inappropriate use of antibiotics has led to the development and spread of multi‐drug resistant (MDR) bacteria, posing a significant threat to human health.^[^ ^1^, ^2^, ^3^ ^]^ A plethora of new antibacterial agents have been developed to combat multi‐drug resistant bacterial infections.^[^ ^3^, ^4^, ^5^, ^6^, ^7^ ^]^ Despite effective treatment regimens, the concern of promoting bacterial resistance during long‐term use of antibiotics remains. Alternatively, photodynamic inactivation (PDI), which rely on highly reactive oxygen species (ROS) to cause oxidative stress to bacteria, has been shown to be more effective in treating MDR bacteria than traditional chemotherapy regimens, with negligible risk of inducing drug resistance.^[^ ^8^, ^9^, ^10^, ^11^, ^12^, ^13^ ^]^

Organic and organometallic photosensitizers (PSs) are widely used as PDI reagents due to their capability to produce ROS.^[^ ^14^, ^15^, ^16^, ^17^, ^18^, ^19^, ^20^, ^21^, ^22^, ^23^, ^24^, ^25^ ^]^ Owing to the many derivatization possibilities, a handful “tailor‐made” organic PSs have been synthesized and displayed promising anticancer and antibacterial efficiency.^[^ ^21^, ^26^, ^27^, ^28^ ^]^ Regardless of the successful PDI performance of some organic PSs, a large portion of them display quenched fluorescence, and poor ROS generating capability due to their aggregation tendency in physiological environments.^[^ ^9^, ^29^, ^30^, ^31^, ^32^, ^33^, ^34^, ^35^, ^36^ ^]^ Heavy metal centers (Pt and Ru) in organometallic PSs have been documented to facilitate intersystem crossing (ISC) processes, leading to enhanced ROS production.^[^ ^37^, ^38^ ^]^ In addition, Pt^II^ PSs exhibited merit of facile photophysical properties tuning by simply coordination with suitable ligands. Currently, Pt^II^ PSs are mainly been applied in destruction of cancer cells and showed promising outcomes.^[^ ^39^, ^40^, ^41^, ^42^ ^]^ Whereas, the use of Pt^II^ PSs (typically in form of metallacycles and metallacages) for antibacterial purposes remains in its infancy. For instance, Sun and coworkers reported a metallacycle‐based supramolecular PSs system for image‐guided photodynamic inactivation of *Escherichia coli* (∼30% efficacy) and *Staphylococcus aureus* (≈70% efficacy) with 450 nm light irradiation (36 J cm^−2^) at a concentration of ≈2 µM.^[^ ^42^ ^]^ It has been well described that PSs with bacterial membrane targeting capability generally display promising antibacterial performance due to their efficient damage to the bacterial membrane by ROS.^[^ ^43^, ^44^, ^45^, ^46^ ^]^ In order to achieve membrane targeting, a sophisticated fabrication process is typically required for Pt^II^ PSs. Niu and coworkers synthesized a bacterial membrane‐anchoring PS by decoration of organo‐Pt^II^ metallacycle with tobacco mosaic virus coat protein and TAT peptide. The resulting complex exhibited an efficient inactivation performance towards *E. coli* (96.3% efficacy) and *S. aureus* (70% efficacy) under light irradiation (22.5 J cm^−2^) at a concentration of ≈40 µM.^[^ ^39^ ^]^ Up until now, antibacterial performance of Pt^II^ PSs have not been comparable to that of organic PSs (which are effectively antibacterial in sub‐micromolecular concentrations). Therefore, organometallic PSs with potent activity against antibiotic resistant bacteria are in high demand.

**Scheme 1:**
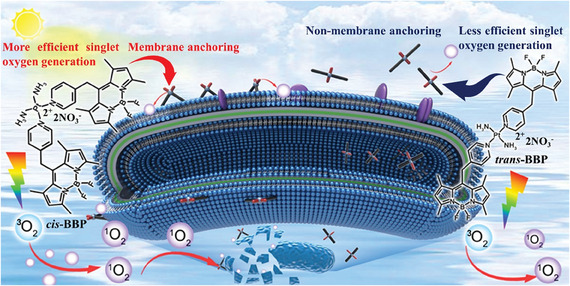
Molecular structure and antibacterial mechanisms of photoactive BODIPY‐Pt^II^ isomers.

The configuration of Pt^II^ determines its anticancer and antibacterial performance, as demonstrated by the classical cis‐ and trans‐platin due to their different abilities of biomolecular interactions.^[^ ^47^, ^48^ ^]^ It has not been addressed whether this phenomenon also exist in Pt^II^ based PSs in bacterial PDI. Herein, we synthesized two Pt^II^ based organometallic PSs (*cis*‐BBP and *trans*‐BBP) by attaching a BODIPY‐pyridine segment to a *cis‐* and *trans*‐Pt^II^ precursor, respectively. *cis*‐BBP exhibited superior singlet oxygen generation efficiency and bacterial membrane intercalation ability over the counter‐part *trans*‐BBP. Correspondingly, *cis*‐BBP exhibited remarkable PDI efficiency (> 99.9%) in MDR *A. baumannii* (≈400 nM, 12 J cm^−2^) and *S. aureus* (≈100 nM, 18 J cm^−2^). In the case of *trans*‐BBP, 6 and 15‐fold of concentration and higher light doses (30 J cm^−2^ for MDR *A*. *baumannii*) were required to achieve the same effect. To the best of our knowledge, *cis*‐BBP is the first organo‐Pt^II^ broad‐spectrum antibacterial PS effective at sub‐micromolecular concentrations. Furthermore, we demonstrate for the first time that the configuration of the Pt^II^ center in organometallic PS largely influences the antibacterial efficiency (**Scheme** **1**).

## 2 Results and Discussion

### 2.1 Syntheses and Structural Characterization

**Figure 1:**
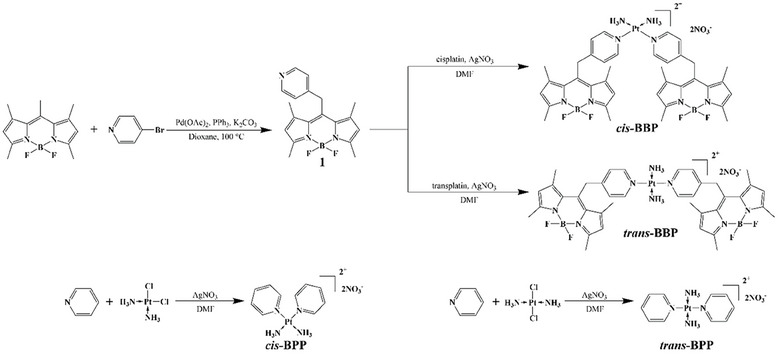
Synthetical route for *cis*‐BBP, *trans*‐BBP, *cis*‐BPP and *trans*‐BPP.

The synthetic route for *cis*‐BBP and *trans*‐BBP together with Pt^II^ complexes without BODIPY (*cis*‐BBP and *trans*‐BBP) are shown in **Figure** **1**. The pyridine‐bearing ligand 1 was synthesized using pentamethyl‐BODIPY as a starting material followed by a reported Pd catalyzed C(_sp_^3^)‐H activation on BODIPY.^[^ ^49^ ^]^ The obtained pyridine attached BODIPY was then coordinated with silver nitrate activated cisplatin and transplatin to yield *cis*‐BBP (18.10%) and *trans*‐BBP (24.30%), respectively. The control complexes *cis*‐BBP and *trans*‐BBP were synthesized by coordination between pyridine and silver nitrate activated cisplatin and transplatin, respectively. The yields for *cis*‐BBP and *trans*‐BBP were 68.70% and 73.00%, respectively. The detailed characterizations, including ^1^H NMR, ^13^C NMR and high‐resolution mass spectra are shown in the supporting information (Figure S22–S33, Supporting Information). Cisplatin showed a slightly lower‐fielded proton δ (coordinated N*H*_3_) compared to that of transplation.^[^ ^50^ ^]^ The δ of N*H*_3_ in *cis*‐BBP and *trans*‐BBP was 4.73 and 4.62 ppm in deuterated DMSO (2.50 ppm as reference). The proton δ of methylene bridge (C*H*_2_) of *cis*‐BBP and *trans*‐BBP was 4.47 and 4.56 ppm (in deuterated DMSO), respectively (Figure S1, Supporting Information). Correspondingly, the proton δ of N*H*_3_ of *cis*‐BBP and *trans*‐BBP was 4.73 and 4.62 ppm (in deuterated DMSO), respectively. The similar tendency of proton δ pattern coordinated N*H*_3_ suggests the desired configuration of synthesized Pt^II^ complexes. The photostability and chemical stability of synthesized complexes in physical environment was characterized by fluorescent spectroscopy and HPLC (Figure S2–S4, Supporting Information).

### 2.2 Theoretical Prediction of Photochemical Properties of Synthesized Complexes

**Figure 2:**
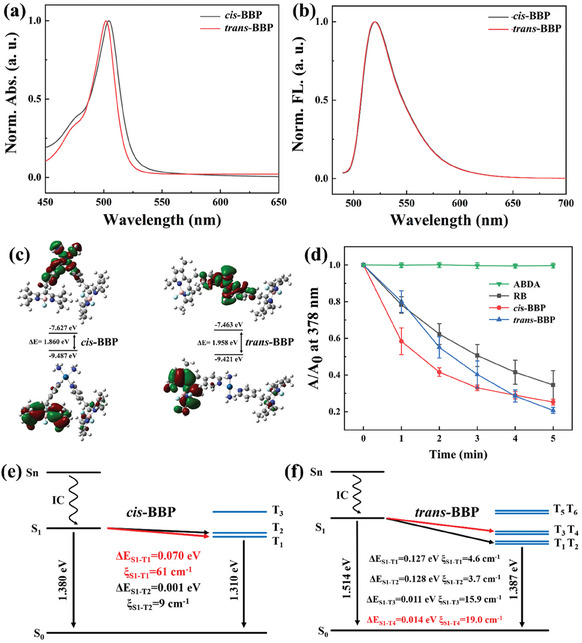
a) and b) Absorption and fluorescent emission spectra of *cis*‐BBP and *trans*‐BBP. c) Frontier molecular orbital amplitude plots and electronic properties of *cis*‐BBP and *trans*‐BBP. d) Time dependent ^1^O_2_ generation ability of RB, *cis*‐BBP and *trans*‐BBP [2.5 µM] tested using ABDA [50 µM] as an indicator under white light irradiation (100 mW cm^−2^). e) and f) Energy level diagrams and calculated ξ_ST_ between different singlet‐triplet channels for *cis*‐BBP and *trans*‐BBP.

*Cis*‐BBP and *trans*‐BBP displayed UV‐vis and fluorescent emission profile resembles that of regular BODIPY (**Figure** **2**a,b). The absorption and fluorescent emission maxima centered at 500 nm and 520 nm for *cis*‐BBP and 503 nm and 520 nm for *trans*‐BBP, respectively. This slightly different absorption maxima for *cis*‐BBP and *trans*‐BBP suggested that coordination with different configurations of Pt^II^ center subtly influence the electronic transitions of the complexes. In addition, the fluorescent quantum yields for *cis*‐BBP and *trans*‐BBP were determined to be 34.25% and 79.74% in water using Rhodamine B (96%) as reference.

The frontier molecular orbital amplitude plots revealed the LUMOs was located at the Pt^II^‐pyridinium segments and HOMOs was located in the BODIPY segments in both synthesized Pt^II^ PSs. The calculated HOMO‐LUMO energy gaps for *cis*‐BBP and *trans*‐BBP were 1.860 and 1.958 eV, respectively (Figure 2c). The slightly different energy gaps were believed to be due to different configurations of the Pt^II^ center. This might explain the slightly different absorption maxima of the two PSs. Photo‐assisted ^1^O_2_ production efficiency of *cis*‐BBP, *trans*‐BBP and Rose‐Bengal (RB) were characterized using the corresponding sensor 9,10‐Anthracenediyl‐bis(methylene)dimalonic acid (ABDA).^[^ ^51^, ^52^ ^]^ As shown in Figure 2d, light irradiation until 5 min did not cause obvious absorbance reduction at 378 nm of ABDA. Whereas *cis*/*trans*‐BBP and RB resulted in absorbance reduction of ABDA at 378 nm in a time‐dependent manner under light irradiation within 5 min, indicating generation of ^1^O_2_. The absorbance reduction of ABDA in the presence of RB upon exposed to light irradiation for 5 min was less significant comparing to that of both synthesized Pt^II^ complexes (≈67% absorbance reduction of ABDA versus ≈80% absorbance reduction for synthesized PSs). Upon light irradiation for 1 min, *cis*‐BBP caused ≈42% reduction of ABDA absorbance at 378 nm, whereas the corresponding value for *trans*‐BBP was ≈20%. The unequal absorbance persisted until light irradiation for 4 min, when the two Pt^II^ complexes caused a similar ≈71% absorbance reduction of ABDA. When light irradiation time amounted to 5 min, ^1^O_2_ generation efficiency (Φ) for *cis*‐BBP and *trans*‐BBP was calculated to be 74.22% and 44.64% according to the below equation, respectively.

(1) $$\Phi \;=\left(\Phi_{\mathrm{RB}}\times \;\frac{K\times A_{\mathrm{RB}}}{K_{\mathrm{RB}}}\times A\right)\;\times 100\%$$

Where Φ_RB_ stands for the quantum efficiency of ^1^O_2_ of standard reference Rhodamine B. K and K_RB_ stand for the decomposition rate of ABDA in the presence of Pt‐complexes and Rhodamine B, respectively. A and A_RB_ stand for the absorbance at 378 nm of ABDA in the presence of Pt‐complexes and Rhodamine B at the time point light irradiation for 5 min. The detailed UV‐vis spectra of ABDA itself and in the presence of three compounds under light after time‐dependent light irradiation are shown in Figure S5 (Supporting Information).

In order to explain the enhanced ^1^O_2_ generation yield for *cis*‐BBP, theoretical calculations were performed (Figure 2e,f). The oscillator factors (*f*), vertical excited energies, wavelengths, together with spin orbit coupling constants between the lowest singlet exited (S_1_) and different triplet states for both *cis*‐BBP and *trans*‐BBP are shown in Table S1–S3 (Supporting Information). For *cis*‐BBP, the maxima *f* was calculated to be 0.0334 corresponding to S_0_‐S_10_ transition with excitation wavelength of 514.77 nm. This matched the UV‐vis absorption maxima with a wavelength of 500 nm. According to the Kasha rule, it was reasonable to conclude that *cis*‐BBP would undergo a quick internal conversion (IC) process to yield the lowest locally‐excited (LE) state S_1_ after excitation.^[^ ^53^ ^]^ The spin‐orbit coupling constant between the lowest singlet excited state (S_1_) to different triplet states (ξ_S1‐T1_, ξ_S1‐T2_, and ξ_S1‐T3)_ was calculated to be 60.66, 9.03, and 27.47 cm^−1^, respectively. The corresponding energy band gap between singlet and triplet states (ΔE_S1‐T1_, ΔE_S1‐T2_, and ΔE_S1‐T3_) were calculated to be 0.070, 0.001, and −0.091 eV, respectively. The negative ΔE_S1‐T3_ suggested a higher energy of theT_3_ state, and the intersystem crossing (ISC) would therefore be forbidden. The pathway of S_1_ to T_1_ constituted the dominant ISC route to yield ^1^O_2_ considering the high spin‐orbit coupling constant (Figure 2e). In the case of *trans*‐BBP, excitation to S_7_ (estimated wavelength of 540.87 nm) was likely to happen (maximum *f* of 0.1146). And the high excited state transformed to the lowest S_1_ state through a quick IC process. The ξ_S1‐T1_, ξ_S1‐T2_, ξ_S1‐T1_, and ξ_S1‐T4_ was calculated to be 4.66, 3.68, 15.92, and 18.99 cm^−1^, respectively. The ΔE_S1‐T1_, ΔE_S1‐T2_, ΔE_S1‐T3_, and ΔE_S1‐T4_ was calculated to be 0.127, 0.128, 0.011, and 0.014 eV (Figure 2f). The likely ISC pathway was S_1_‐T_4_ according to the description of rate for ISC (*k*_isc_):

(2) $$k_{isc}\propto \frac{\xi_{ST}^{2}}{exp^{\left(\Delta E_{ST}^{2}\right)}}$$

where, ξ_ST_ and ΔE_ST_ stands for spin‐orbit coupling constant and energy gap between singlet and triplet states.^[^ ^54^ ^]^ Therefore, these theoretical calculated results explained the relatively high efficiency of sensitizing ^3^O_2_ to yield ^1^O_2_ of *cis*‐BBP as compared to *trans*‐BBP.

### 2.3 Bacterial Membrane Anchoring Capability

**Figure 3:**
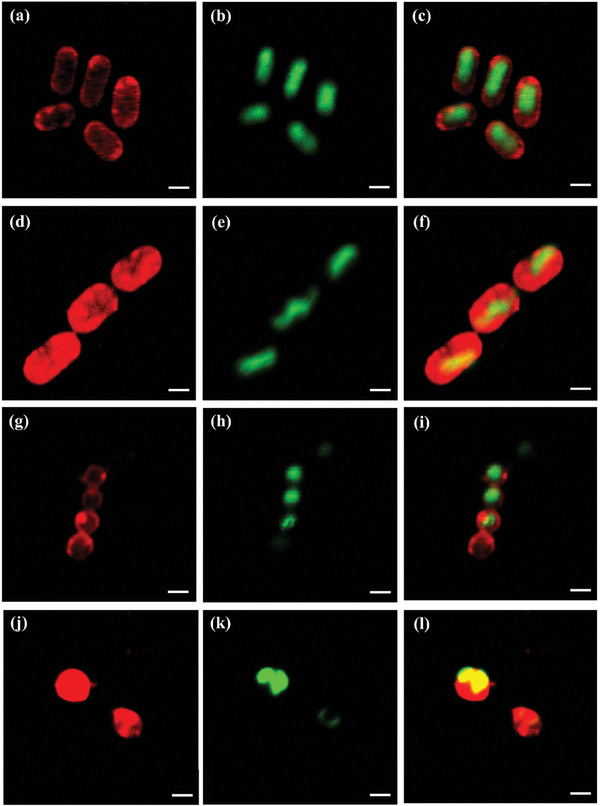
CLSM images of MDR *A. baumannii* and MRSA stained with PSs and DAPI. a) MDR *A. baumannii* stained with *cis*‐BBP shown in red‐false color. b) MDR *A. baumannii* stained with DAPI shown in green‐false color. c) merge of (a) and (b). d) MDR *A. baumannii* stained with *trans*‐BBP shown in red‐false color. e) MDR *A. baumannii* stained with DAPI shown in green‐false color. f) merge of (d) and (e). g) *S. aureus* stained with *cis*‐BBP shown in red‐false color. h) *S. aureus* stained with DAPI shown in green‐false color. i) merge of (g) and (h). j) *S. aureus* stained with *trans*‐BBP shown in red‐false color. k) MDR *A. baumannii* stained with DAPI shown in green‐false color. (l) merge of (j) and (k). Excitation wavelengths for PSs and DAPI were 488 and 405 nm, respectively. [PSs] = 3.00 µg mL^−1^, [DAPI] = 3.30 µg mL^−1^, scale bar = 1 µm.

Owing to the photoluminescent properties of the photosensitizers, we employed confocal laser scanning microscopy (CLSM) to investigate the interactions between *cis*‐BBP and *trans*‐BBP, and multi‐drug resistant *Acinetobacter baumannii* (a gram‐negative bacteria) and MRSA (a gram‐positive bacteria). We used 4′,6‐diamidino‐2‐phenylindole (DAPI) to stain the bacterial DNA (green false color). The false red color emission from *cis*‐BBP was mainly MRSA (**Figure** **3**g–i). In contrast, DAPI stained the interior area of MDR *A. baumannii* and MRSA. This suggested *cis*‐BBP acted as an effectively bacterial membrane anchoring photosensitizer. In contrast, when using *trans*‐BBP a large portion of red‐green false color overlay could be observed in both bacterial species (Figure 3d–f,j–l). This indicated *trans*‐BBP might not be a promising bacterial membrane anchoring agent comparing to *cis*‐BBP. The DIC photos of stained bacteria were shown in Figure S6 (Supporting Information). Furthermore, ζ potentials of MDR *A. baumannii* and MRSA were measured to be −14.97 ± 1.68 and −15.50 ± 2.12 mV, respectively. The ζ potentials of the two bacterial species in the presence of 2.00 µg mL^−1^ *cis*‐BBP increased20 to −1.69 ± 0.65 and −3.53 ± 1.88 mV for MDR *A. baumannii* and MRSA. The corresponding values in the presence of 2.00 µg mL^−1^ *trans*‐BBP were −9.32 ± 0.67 and −8.76 ± 0.71 mV, respectively (Table S4, Supporting Information). This further indicated that *cis*‐BBP could more efficiently bind to bacterial membrane.

### 2.4 Bacterial Membrane Binding Mechanism

**Figure 4:**
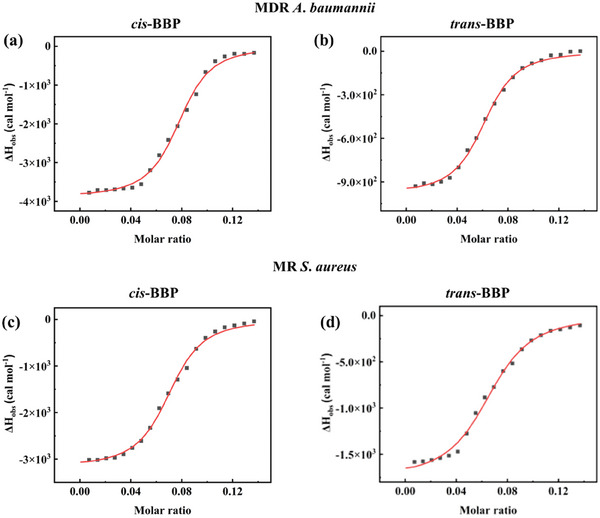
ITC titration data of *cis*‐BBP a) and *trans*‐BBP b) treated MDR *A*. *baumannii*, and *cis*‐BBP c) and *trans*‐BBP d) treated MRSA.

Isothermal titration calorimetry (ITC) assay revealed detailed binding mechanisms of Pt^II^ complexes towards both bacterial membranes. The negative enthalpy change (ΔH_b_) of Pt^II^ complexes in MDR *A. baumannii* (−3.89 ± 0.055 kcal mol^−1^ for *cis*‐BBP and −0.98 ± 0.013 kcal mol^−1^ for *trans*‐BBP) and MRSA (−3.17 ± 0.044 kcal mol^−1^ for *cis*‐BBP and −1.76 ± 0.035 kcal mol^−1^ for *trans*‐BBP) suggested electrostatic interactions and hydrogen bonding contributed to bacterial binding (**Figure** **4**; Table S5, Supporting Information).^[^ ^24^ ^]^ The more negative ΔH_b_ suggested more pronounced electrostatic and hydrogen bonding interactions of *cis*‐BBP in individual bacterial species. In addition, positive entropy change (ΔS_b_) suggested hydrophobic interactions also contributed to PtII complexes to bacterial membrane (Table S5, Supporting Information).^[^ ^24^ ^]^ The detailed TΔS_b_ values of Pt^II^ complexes on MDR *A. baumannii* (4.59 kcal/mol for *cis*‐BBP and 7.36 kcal mol^−1^ for *trans*‐BBP) and MRSA (5.19 kcal mol^−1^ for *cis*‐BBP and 6.26 kcal mol^−1^ for *trans*‐BBP) indicated hydrophobic interactions are dominating interactions between *trans*‐BBP and both bacteria strains.

### 2.5 In Vitro Photo‐assisted Antibacterial Activity

**Figure 5:**
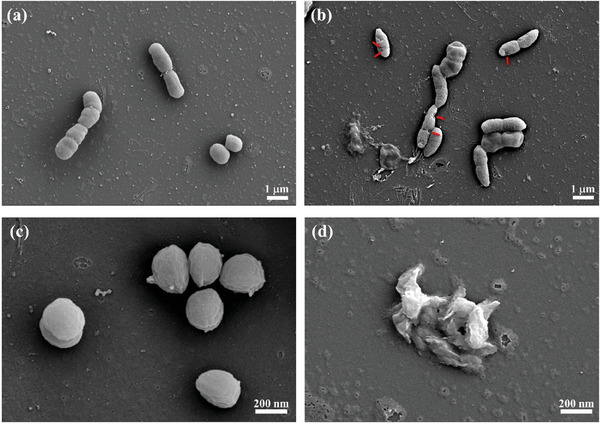
Morphology of MDR *A. baumannii* in the absence of *cis*‐BBP a) and in the presence of *cis*‐BBP after light irradiation b). Morphology of *S. aureus* in the absence of *cis*‐BBP c) and in the presence of *cis*‐BBP after light irradiation for d). [*cis*‐BBP] = 0.40 µg mL^−1^, light dose 30 J cm^−2^.

It has previously been reported that antibacterial PSs with membrane anchoring capability generally display promising antibacterial activity.^[^ ^46^ ^]^ We first investigated the light‐assisted bacterial antimicrobial activity of the synthesized PSs by scanning electronic microscopy (SEM). As shown in Figure 4a MDR *A. baumannii* has a smooth surface without treatment with *cis*‐BBP. After light irradiation in the presence of *cis*‐BBP, obvious damage to the cellular membrane and bacterial cell lysis could be seen (**Figure** **5**b). *S. aureus* underwent substantial cell lysis after light irradiation, potentially suggesting more severe damage to the bacterial cell wall (Figure 5c,d).

**Figure 6:**
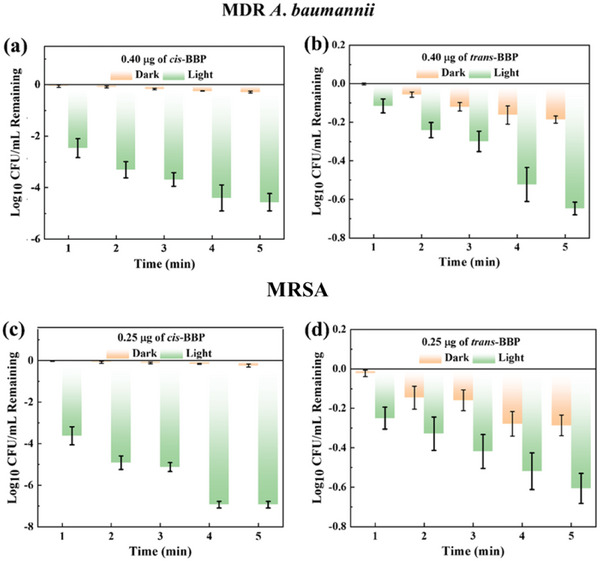
Antibacterial activity of *cis*‐BBP and *trans*‐BBP in dark and under light irradiation with different concentrations over time. Incubation time‐dependent MDR *A. baumannii* bactericidal activity of *cis*‐BBP a) and *trans*‐BBP (b) in dark and under light irradiation. [Pt^II^ complexes] = 0.40 mg mL^−1^. Incubation time‐dependent MRSA bactericidal activity of *cis*‐BBP c) and *trans*‐BBP d) in dark and under light irradiation. [Pt^II^ complexes] = 0.25 mg mL^−1^.

Encourage by these results, further testing of antibacterial activity of both PSs on MDR *A. baumannii* and MRSA was performed. The best MDR A. *baumannii bactericidal* activity was 47.98% (**Figure** **6**a; Table S6, Supporting Information) for *cis*‐BBP with a final concentration of 0.40 µg mL^−1^ (≈400 nM) and incubation for 5 mins without light irradiation. Comparable MDR A. baumannii bactericidal activity in the presence of *trans*‐BBP (49.35% and 48.14%, Table S7, Supporting Information) was achieved with final concentrations of 1.00 and 1.50 µg mL^−1^ (> 2.50 folds concentration comparing that of *cis*‐BBP) after 5 mins incubation, respectively (Table S7, Supporting Information). Upon incubation with higher concentrations of *trans*‐BBP (2.00 and 2.50 µg mL^−1^) for 5 min, the bactericidal activity was 53.31 and 59.51%, respectively (Table S7, Supporting Information). The similar dark MDR *A. baumannii* bactericidal activity obtained with more diluted dispersion of *cis*‐BBP suggests that bacterial membrane anchoring could be one of the determining mechanisms in bacterial killing even in dark conditions.

Upon light irradiation in the same condition (concentration of Pt‐complexes of 0.40 µg mL^−1^), *cis*‐BBP exhibited robust anti‐MDR *A. baumannii* activity against *trans*‐BBP (Figure 6a,b). The CFU remaining (in form of log10) in the presence of *cis*‐BBP gradually dropped to −4 (antibacterial activity of ≈99.99%) with increasing light irradiation time dose from 6 to 30 J cm^−2^ (Figure 5a). Limited bactericidal activity was observed without light irradiation. In the case of *trans*‐BBP, the minimum CFU remaining (in form of log10) was −0.6 (≈antibacterial activity of ≈74.89%, Figure 6b). Comprehensive light assisted bactericidal activity in varied complex concentration and light irradiation dose was evaluated. *Cis*‐BBP exhibited limited anti‐MDR *A. baumannii* activity with a treatment of light irradiation at 0.05 µg mL^−1^ concentrations (maxima efficiency was 58.01% at a light dose of 30 J cm^−2^, Figure S7 and Table S8, Supporting Information). When concentrations reached 0.10 µg mL^−1^ (≈100 nM), more efficient bactericidal activity was achieved using light irradiation doses of 12, 18, 24, and 30 J cm^−2^ (corresponding efficiency was 92.29%, 94.50%, 96.35%, and 97.79%, Table S8, Supporting Information). In the presence of 0.20 µg mL^−1^ (≈200 nM), > 99% of bacteria were killed with a light irradiation dose of 24 and 30 J cm^−2^ (99.40% and 99.63%, respectively). In order to reach similar bactericidal activity (99.29%), a higher concentration of *trans*‐BBP (2.50 µg mL^−1^, ≈2.50 µM) was required under higher light irradiation (30 J cm^−2^, Figure S8 and Table S9, Supporting Information). In lower concentrations (2.00, 1.50, and 1.00 µg mL^−1^), *trans*‐BBP displayed inferior light‐assisted antibacterial activity comparing to that of *cis*‐BBP (Figure S8 and Table S9, Supporting Information). As discussed above, we believe both ^1^O_2_ generation and bacterial membrane anchoring capability could explain these different PDI performances. Photographs of MDR *A. baumannii* colonies reflected the antibacterial activity of *cis*‐BBP and *trans*‐BBP in dark and light irradiation (Figure S9, Supporting Information). In contrast, Pt^II^ complexes without BODIPY, namely *cis*‐BPP and *trans*‐BPP showed minimal antibacterial activity against MDR *A. baumannii* without light irradiation (below 10% at the highest concentration after incubation for 5 min, Figure S10 and S11, Supporting Information). Under light irradiation (30 J cm^−2^), both control complexes displayed some anti‐ MDR *A. baumannii* activity at the corresponding highest concentrations (*cis*‐BPP ≈62% and *trans*‐BPP ≈53%, Figure S10 and S11, Supporting Information). The improved antibacterial activity of *cis*‐BPP and *trans*‐BPP under light irradiation suggested that the two control Pt^II^ complexes might have some ^1^O_2_ generating capability.

In addition to MDR *A. baumannii*, the bactericidal activity of Pt‐complexes on gram positive MRSA was investigated. As shown in Figure 6c, cis‐BBP displayed moderate anti MRSA activity without light irradiation (maxima bacterial killing efficiency of 42.08% with incubation at highest concentrations of 0.25 µg mL^−1^ for 5 min, Figure 6c; Table S10, Supporting Information). Similar dark antibacterial activity against MRSA was obtained using high concentrations of *trans*‐BBP (1.00, 1.50, and 2.00 µg mL^−1^, Table S11, Supporting Information). Relatively low concentrations of *trans*‐BBP (0.50 µg mL^−1^, ≈500 nM) yielded lower anti‐MRSA activity (29.34% after 5 min incubation, Figure S12 and Table S11, Supporting Information). The highest MRSA bactericidal activity (47.96%) of *trans*‐BBP were obtained at the highest tested concentration of 2.00 µg mL^−1^ (8‐fold higher concentration comparing to that of *cis*‐BBP) after incubation for 5 min. Compared to MDR *A. baumannii*, MRSA was more sensitive towards treatment of *cis*‐BBP under white light irradiation due to its different membrane structure.^[^ ^55^ ^]^ Similarly, the *cis*‐BBP exhibited more powerful light assisted antibacterial capability in contrast to trans‐BBP. 0.25 µg mL^−1^ of cis‐BBP and light dose of 30 J cm^−2^ could result nearly 100% MRSA killing (CFU remaining (in form of log10) of −7, Figure 6c). Under the identical condition, trans‐BBP only caused CFU remaining of −0.6 (antibacterial activity of ≈74.89%, Figure 6d). More detailed photo‐assisted antibacterial performance was evaluated. Upon white light irradiation for 1 min (6 J cm^−2^), >99% MRSA was killed in the presence of *cis*‐BBP (final concentrations of 0.15, 0.20, and 0.25 µg mL^−1^, Figure S13 and Table S12, Supporting Information). With prolonged light irradiation (5 min), the MRSA bactericidal activity reached 99.30% even in the presence of only 0.05 µg mL^−1^ (≈50 nM) of *cis*‐BBP. Nearly 100% bacterial killing were observed in the presence of 0.25 µg mL^−1^ (≈250 nM) of *cis*‐BBP with light doses of 24 and 30 J cm^−2^. More concentrated *trans*‐BBP (1.50 and 2.00 µg mL^−1^, ≈1.50 and 2.00 µM) was required for efficient MRSA killing (> 99%) under white light irradiation for at least 1 min (Figure S12 and Table S13, Supporting Information). Diluted *trans*‐BBP yielded reduced anti‐ MRSA bactericidal activity in a light irradiation time‐dependent manner (38.86 to 74.48% and 86.78% to 96.71% in the presence of 0.50 and 1.00 µg mL^−1^, respectively, Table S13, Supporting Information). Photographs of MRSA colonies in the presence of *cis*‐BBP and *trans*‐BBP in dark environment and light irradiation revealed a similar of antibacterial activity (Figure S14, Supporting Information). The anti‐MRSA activity of *cis*‐BPP and *trans*‐BPP resembles that of MDR *A. baumannii* (maximum killing effect of ≈10% in dark and ≈54% under light irradiation in highest concentration and longest incubation time, Figure S15 and S16, Supporting Information). *cis*‐BBP displayed lower 50% minimum inhibitory concentration (MIC_50_) comparing to *trans*‐BBP in MDR *A. baumannii* 1 and MRSA at experimental light irradiation doses (**Table** **1**). The values of *cis*‐BBP ranged from 0.095 µg mL^−1^ to 0.048 µg mL^−1^ and 0.031 µg mL^−1^ to 0.025 µg mL^−1^ with increasing light dose from 6 J cm^−2^ to 30 J cm^−2^ for *A. baumannii* and MRSA, respectively. The corresponding values of *trans*‐BBP ranged from 1.60 µg mL^−1^ to 0.70 µg mL^−1^ and 0.66 µg mL^−1^ to 0.43 µg mL^−1^ in the identical light doses for *A. baumannii* and MRSA, respectively. The lowest MIC_50_ ratio between *trans*‐BBP and *cis*‐BBP for MDR *A. baumannii* was 14.58 (30 J cm^−2^) and the highest 22.22 (12 J cm^−2^, Table S14, Supporting Information). In the case of MRSA, the corresponding lowest ratio was 17.20 (30 J cm^−2^) and highest 21.29 (6 J cm^−2^, Table S14, Supporting Information). The light assisted MIC_90_ values of *cis*‐BBP and *trans*‐BBP also reduced with increasing light dose for *A. baumannii* and MRSA (Table 1). The lowest values of *cis*‐BBP for *A. baumannii* and MRSA were 0.088 µg mL^−1^ and 0.045 µg mL^−1^, respectively. The corresponding values of *trans*‐BBP for *A. baumannii* and MRSA were 1.32 µg mL^−1^ and 0.84 µg mL^−1^, respectively. Similarly, the lowest MIC_90_ ratio between *trans*‐BBP and *cis*‐BBP for *A. baumannii* was 15.00 (30 J cm^−2^) and the highest 24.02 (12 J cm^−2^, Table S15, Supporting Information). As for MRSA, the corresponding ratios were 18.67 (30 J cm^−2^) and 22.36 (6 J cm^−2^, Table S15, Supporting Information), respectively. The lower concentrations for efficient bacteria killing (>99%) combined with a high MIC_50_/MIC_90_ ratio reflected *cis*‐BBP as generally more potent in photo‐assisted antibacterial activity in both gram‐negative and gram‐positive bacteria as compared to its trans‐isomer. These results might suggest cis‐configurated isomers of organo‐Pt^II^ complexes to be displaying enhanced photodynamic inactivation efficiency towards bacteria as compared to trans‐isomers, because of better photo‐assisted ROS generating and bacterial membrane locating capability of the cis‐isomer.

**Table 1:** MIC_50_ and MIC_90_ values of cis‐BBP and trans‐BBP toward MDR *A. baumannii* and MRSA isolates after various dose of light irradiation.

Light dose (J cm^−2^)	MIC_50_ (µg mL^−1^)				MIC_90_ (µg mL^−1^)			
	MDR *A. baumannii*		MRSA		MDR *A. baumannii*		MRSA	
	*cis*‐BBP	*trans*‐BBP	*cis*‐BBP	*trans*‐BBP	*cis*‐BBP	*trans*‐BBP	*cis*‐BBP	*trans*‐BBP
6	0.095	1.60	0.031	0.66	0.181	2.92	0.055	1.23
12	0.054	1.20	0.028	0.55	0.097	2.33	0.051	0.98
18	0.054	0.97	0.027	0.51	0.096	1.85	0.048	0.94
24	0.048	0.85	0.026	0.46	0.089	1.70	0.046	0.88
30	0.048	0.70	0.025	0.43	0.088	1.32	0.045	0.84

### 2.6 In Vivo Photo‐Assisted Antibacterial Activity

**Figure 7:**
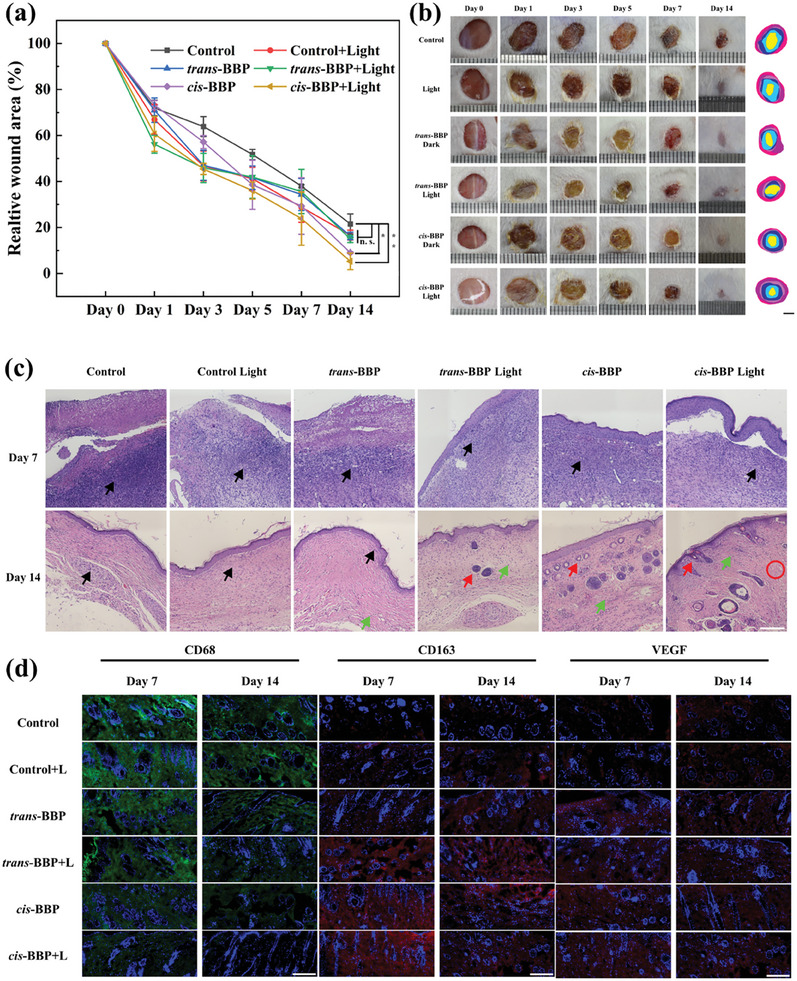
a) Relatively wound surface area of MRSA infected male ICR mice under different treatment on. day 0, 1, 3, 5, 7, and 14. b) Wound photos of corresponding groups on day 0, 1, 3, 5, 7, and 14. c) H&E staining of the infected skin on the day 7 and 14 after different treatments. d) Immunofluorescent photos (CD68, CD163 and VEGF) of MRSA infected mice on day 7 and day 14 with different treatments. [*cis*‐BBP] and [*trans*‐BBP] = 0.10 µg mL^−1^, light irradiation dose was 30 J cm^−2^. Scale bar was 100 µm. Six mice were used in each group and the experiment was repeated for three times. *p< 0.05, **p< 0.01.

Based on the above in vitro antibacterial activity of both PSs, we further tested their in vivo antibacterial effects. 5 weeks male ICR mice with MSRA‐triggered skin infections (bacterial dispersion using OD_600 nm_ = 0.5) were used as in vivo models. The dark groups referred to those with addition of 0.9% NaCl (control), *cis*‐BBP and *trans*‐BBP (50 µL, 0.10 µg mL^−1^) in the wound without light irradiation, respectively. The light groups referred to the corresponding additions as in the dark groups but with addition of light irradiation of 30 J cm^−2^. As shown in **Figure** **7**a,b, the dark control group exhibited least wound recovery (21.53±4.36% wound surface area remaining on the 14th day of the experiment). In contrast, the corresponding wound remaining ratio of light control was 16.69±2.14%, indicating slightly effect of accelerating wound recovery. In the presence of *trans*‐BBP, the corresponding ratio was 16.32±1.63% (dark) and 14.99±1.55% (light), respectively. The most effective wound healing was observed in the presence of *cis*‐BBP (9.01±0.42% in dark and 5.27±0.44% under light irradiation, Table S16, Supporting Information, respectively.) This indicated a potent wound recovery performance of *cis*‐BBP under light irradiation. In addition, the hematoxylin and eosin (H&E) stained tissue histology of the infected wound and adjacent tissues on 7th day of exhibited wide spread of immunogenic cells, indicating occurrence of inflammatory response (Figure 7c). On 14th day, immunogenic cells could still be observed in dark control, light control and *trans*‐BBP dark groups, but almost not seen in the rest three groups. To note, fibroblast was seen in both Pt^II^ treatment groups (dark and light). Follicle has shown up in *trans*‐BBP dark, *cis*‐BBP dark and light groups. Collagen was found in *cis*‐BBP light group. Furthermore, we evaluated the expression of inflammatory and angiogenesis related biomarkers (CD68, CD163 and VEGF). As shown in Figure 7d; Figure S17 (Supporting Information), treatment of *cis*‐BBP with light irradiation caused least expression of pro‐inflammatory biomarker (CD68) on 7th and 14th days. The most pronounced expression of anti‐inflammatory biomarker (CD163) was found in *cis*‐BBP with light irradiation on 7th day. On 14th day, CD163 was less expressed in *cis*‐BBP light group, which might suggest finish of the inflammatory reaction (Figure 7d and Figure S17, Supporting Information). Blood vessels started to form in the presence of Pt^II^ complexes on 7th day as characterized by VEGF expression (Figure 7d; Figure S17, Supporting Information). Taken together, these results reflected the *cis*‐isomer as being more capable of in vivo antibacterial activity and effective in improving healing of infected wound. To the best of our knowledge, *cis*‐BBP is the first organo‐Pt^II^ photosensitizer with membrane anchoring capability effective against both gram‐negative gram‐positive bacteria at sub‐micromolar concentrations (Table S17, Supporting Information).

### 2.7 In vivo Biocompatibility

Both complexes displayed no observable 24 h dark toxicity in mouse fibroblast L929 cell line at the concentration range of 0.01 to 0.40 µg mL^−1^. Upon light irradiation for 5 min (30 J cm^−2^), both the complexes displayed no photo cytotoxicity below 0.05 µg mL^−1^ (Figure S18, Supporting Information). Further increasing concentration to 0.40 µg mL^−1^ caused certain photocytotoxicity (cell viability of ≈60%, Figure S18, Supporting Information). Despite the cellular photo‐cytotoxicity, the *cis*‐BBP was more efficient in bacterial killing when same concentration and light dose was applied. Besides, application of Pt^II^ PSs caused no obvious body weight and blood parameters changes during the therapy process (Figure S19 and S20, Supporting Information). Moreover, histological analysis revealed no histopathological lesions and abnormalities of major organs (Figure S21, Supporting Information). These results suggested acceptable biocompatibilities of synthesized Pt^II^ PSs.

## 3 Conclusion

In conclusion, the synthesized *cis*‐BBP exhibited more pronounced singlet oxygen production efficiency over the configurational isomer *trans*‐BBP. As suggested by theoretical calculations, the *cis*‐BBP had comparable ΔE_ST_ and enhanced ξ_ST_ as compared to *trans*‐BBP, which allowed the occurrence of efficient ISC. In addition, *cis*‐BBP was more prone to anchor to the membrane of both MDR *A*. *baumannii* (gram‐negative) and *S*. *aureus* (gram‐positive) over *trans*‐BBP. Correspondingly, *cis*‐BBP displayed more efficient in vitro and in vivo antibacterial activity as compared to *trans*‐BBP, requiring low concentrations and light dose. In addition, *cis*‐BBP is the first Pt^II^ based PS that achieve efficient bactericidal activity (>99.9% in both gram‐negative and gram‐positive bacteria) in sub‐micromolar concentration using relatively low light dose (maximum 30 J cm^−2^) with acceptable cytotoxicity. Comparing to previously reported organo‐Pt^II^ PSs, an overall more than 100 times antibacterial activity was obtained for *S*. *aureus* (Table S16, Supporting Information).^[^ ^45^ ^]^ These results suggested *cis*‐BBP is a potent Pt^II^ PSs with apparent broad‐spectrum antibacterial activity. More importantly we have demonstrated the cis/trans configuration of Pt^II^ photosensitizer played significant impact on the antibacterial performance for the first time. We believe this is an important proof of concept for designing potent antibacterial photosensitizers in future.

## 4 Experimental Section

### Materials and Methods

All chemicals were purchased from Macklin Inc. and Sinopharm, and used without further purification. Milli‐Q water (18.2 MΩ) was provided by Direct‐Q water purification system (Millipore Corporation). ^1^H and ^13^C NMR spectra were recorded on Agilent DD2 (300 MHz) and Avance 600 spectrometers. Chemical shifts were reported in parts per million referenced with respect to residual solvent CDCl_3_ = 7.27 ppm, for ^1^H NMR and CDCl_3_ = 77.0 ppm for ^13^C NMR. UV‐vis and fluorescence spectra were recorded using Perkin Elmer Lamda 650 and Itachi F‐7000 spectrometers. High‐resolution mass spectra were recorded on Bruker Dalton maXis spectrometer with Electrospray ionization source. Confocal laser scanning microscopy images was obtained using Carl‐Zeiss LSM 880NLO with excitation wavelength of 488 nm. SEM images of bacteria was obtained on Itachi FESEM (S‐4800II). ζ‐potential of bacteria before and after treated with *cis*‐BBP and *trans*‐BBP was collected using Malvern ZEN 3690. Pentamethyl‐BODIPY was synthesized according to literature using 2,4‐dimethylpyrole acetyl chloride as starting materials.^[^ ^56^ ^]^ Compound 1 was synthesized according to literature using pentamethyl‐BODIPY as starting material through an established Pd(OAc)_2_ catalyzed C(sp^3^)‐H direct arylation process.^[^ ^49^ ^]^ The ^1^H NMR, ^13^C NMR and high‐resolution data of the two compounds were identical with literature.^[^ ^49^ ^]^ The clinical *A. baumannii* strain AB43 was isolated from the affiliated hospital of Yangzhou University in Jiangsu, China. The species was identified using Gram stain, 16S RNA sequencing, and the VITEK‐2 system (bioMerieux, Marcy l'Etoile, France) in the clinical microbiology laboratory. Methicillin‐resistant Staphylococcus aureus (MRSA) was purchased from MINGZHOUBIO with strain no. of ATCC43300.

### Synthesis of *cis*‐BBP

43.40 mg (0.15 mM) of cisplatin was dissolved in dry 2 mL of dry DMF under an inert atmosphere in dark and stirred for 10 min. Subsequently, 51.10 mg (0.30 mM) of AgNO_3_ was added to the mixture and heated at 45 °C for 18 h. Pure Pt^II^ precursor was obtained after removing the precipitation (AgCl) by filtration as yellow solid (45.76 mg, 0.13 mM, 89%). The obtained Pt^II^ precursor was then reacted with 91 mg (0.27 mM) of compound 1 in 2 mL of dry DMF at 55 °C for 16 h under an inert atmosphere in dark. The desired *cis*‐BBP was obtained by precipitation in 20 mL of diethyl ether as orange solid (25.44 mg, 18.2%). ^1^H NMR (400 MHz, DMSO‐*d_6_*) δ: 8.67 (d, J = 6.6 Hz, 4H, ArH), 7.33 (d, J = 6.5 Hz, 4H, ArH), 6.25 (s, 4H, ArH), 4.73 (s, 6H, N‐H), 4.47 (s, 4H, CH_2_), 2.45 (s, 12H, CH_3_), 2.03 (s, 12H, CH_3_). ^13^C NMR (100 MHz, DMSO‐*d_6_*) δ: 162.3, 155.0, 152.5, 150.8, 141.4, 137.5, 131.6, 125.5, 122.2, 35.8, 32.1, 30.8, 15.4, 14.2. IR (KBr) υ: 3429, 3103, 1657, 1622, 1552, 1508, 1307, 1200, 1076, 970, 827, 698, 480 cm^−1^; MS(m/z): HRMS (ESI) Calcd. for C_38_H_46_B_2_F_4_N_10_O_6_Pt ([M‐2NO_3_^−^]^2+^): 453.6802, found: 453.6817.

### Synthesis of *cis*‐BPP

120.00 mg (0.40 mM) of cisplatin was dissolved in 4 mL of dry DMF and stirred under an inert atmosphere in dark for 10 min. Then 139.60 mg (0.82 mM) AgNO_3_ was added to the resulting solution and stirred at 45 °C for 18 h under dark. Pure Pt^II^ precursor was obtained after removing the precipitation (AgCl) by filtration as yellow solid (125.31 mg, 0.36 mM, 89%). The obtained Pt^II^ precursor was then reacted with 85 mg (1.08 mM) of pyridine in 2 mL of dry DMF at 55 °C for 16 h under an inert atmosphere in dark. The desired *cis*‐BPP was obtained by precipitation in 20 mL of diethyl ether as white solid (123.33 mg, 67.8%). ^1^H NMR (400 MHz, DMSO‐*d_6_*) δ: 8.98‐8.67 (d, 4H, ArH), 8.10 (t, 2H, ArH), 7.68‐7.62 (m, 4H, ArH), 4.82 (s, 6H, N‐H). ^13^C NMR (100 MHz, DMSO‐*d_6_*) δ: 152.9, 140.7, 127.4. IR (KBr) υ: 3105, 1612, 1450, 1315, 1149, 1078, 827, 761, 692 cm^−1^; MS(m/z): HRMS (ESI) Calcd. for C_10_H_16_N_6_O_6_Pt ([M‐2NO_3_^−^]^2+^): 193.5506, found:193.5497.

### Synthesis of *trans*‐BBP

43.40 mg (0.15 mM) of transplatin was dissolved in dry 2 mL of dry DMF under an inert atmosphere in dark and stirred for 10 min. Subsequently, 51.10 mg (0.30 mM) of AgNO_3_ was added to the mixture and heated at 45 °C for 18 h. Pure Pt^II^ precursor was obtained after removing the precipitation (AgCl) by filtration as yellow solid (45.76 mg, 0.13 mM, 89%). The obtained Pt^II^ precursor was then reacted with 91 mg (0.27 mM) of compound 1 in 2 mL of dry DMF at 55 °C for 16 h under an inert atmosphere in dark. The desired *trans*‐BBP was obtained by precipitation in 20 mL of diethyl ether as orange solid (34.53 mg, 24.7%). ^1^H NMR (400 MHz, DMSO‐*d_6_*) δ: 8.64 (d, J = 6.5 Hz, 4H, ArH), 7.46 (d, J = 6.5 Hz, 4H, ArH), 6.27 (s, 4H, ArH), 4.61 (s, 6H, N‐H), 4.55 (s, 4H, CH_2_), 2.45 (s, 12H, CH_3_), 2.17 (s, 12H, CH_3_).^13^C NMR (100 MHz, DMSO‐*d_6_*) δ: 155.4, 153.7, 151.1, 142.0, 138.1, 132.1, 126.0, 122.6, 32.7, 16.1, 14.7. IR (KBr) υ: 3153, 3100, 1624, 1547, 1506, 1361, 1304, 1151, 1078, 970, 800, 702 cm^−1^. HRMS (ESI) Calcd. For C_38_H_46_B_2_F_4_N_10_O_6_Pt ([M‐2NO_3_^−^]^2+^): 453.6802, found: 453.6825.

### Synthesis of *trans*‐BPP

120.00 mg (0.40 mM) of transplatin was dissolved in 4 mL of dry DMF and stirred under an inert atmosphere in dark for 10 min. Then 139.60 mg (0.82 mM) AgNO_3_ was added to the resulting solution and stirred at 45 °C for 18 h under dark. Pure Pt^II^ precursor was obtained after removing the precipitation (AgCl) by filtration as white solid (125.31 mg, 0.36 mM, 89%). The obtained Pt^II^ precursor was then reacted with 85 mg (1.08 mM) of pyridine in 2 mL of dry DMF at 55 °C for 16 h under an inert atmosphere in dark. The desired *trans*‐BPP was obtained by precipitation in 20 mL of diethyl ether as white solid (132.80 mg, 73%). ^1^H NMR (400 MHz, DMSO‐*d_6_*) δ: 8.72 (d, 4H, ArH), 8.10 (t, 2H, ArH), 7.78‐7.56 (m, 4H, ArH), 4.65 (s, 6H, N‐H). ^13^C NMR (100 MHz, DMSO‐*d_6_*) δ: 153.2, 140.3, 127.1, IR (KBr) υ: 3158, 3103, 1612, 1457, 1363, 1315, 773, 700 cm^−1^; MS(m/z): HRMS (ESI) Calcd. for C_10_H_16_N_6_O_6_Pt ([M‐2NO_3_^−^]^2+^): 193.5506, found:193.5501.

## Conflict of Interest

The authors declare no conflict of interest.

## Supporting information

Supporting Information

## Data Availability

Research data are not shared.

## References

[1] J. M. A. Blair , M. A. Webber , A. J. Baylay , D. O. Ogbolu , L. J. V. Piddock , Nat. Rev. Microbiol. 2015, 13, 42. 25435309 10.1038/nrmicro3380

[2] M. Ferri , E. Ranucci , P. Romagnoli , V. Giaccone , Crit. Rev. Food. Sci. Nutr. 2017, 57, 2857. 26464037 10.1080/10408398.2015.1077192

[3] S. E. Rossiter , M. H. Fletcher , W. M. Wuest , Chem. Rev. 2017, 117, 12415. 28953368 10.1021/acs.chemrev.7b00283 PMC5869711

[4] H.a?V. Jenssen , P. Hamill , R. E. W. Hancock , Clin. Microbiol. Rev. 2006, 19, 491. 16847082 10.1128/CMR.00056-05 PMC1539102

[5] D. J. Payne , M. N. Gwynn , D. J. Holmes , D. L. Pompliano , Nat. Rev. Drug. Discov. 2007, 6, 29. 17159923 10.1038/nrd2201

[6] K. L. Laplante , A. Dhand , K. Wright , M. Lauterio , Ann. Med. 2022, 54, 1686. 35723082 10.1080/07853890.2022.2085881 PMC9225766

[7] C. Zhang , R. Yu , L. Wang , H. Huang , J. Wang , X. Liao , X. Duan , Y. Xiong , Eur. J. Med. Chem. 2022, 240.

[8] J. P. Celli , B. Q. Spring , I. Rizvi , C. L. Evans , K. S. Samkoe , S. Verma , B. W. Pogue , T. Hasan , Chem. Rev. 2010, 110, 2795. 20353192 10.1021/cr900300p PMC2896821

[9] X. Li , S. Lee , J. Yoon , Chem. Soc. Rev. 2018, 47, 1174. 29334090 10.1039/c7cs00594f

[10] R. An , X. Cheng , S. Wei , Y. Hu , Y. Sun , Z. Huang , H.‐Y. Chen , D. Ye , Angew. Chem., Int. Ed. 2020, 59, 20817. 10.1002/anie.202009141 32686894

[11] J. F. Lovell , T. W. B. Liu , J. Chen , G. Zheng , Chem. Rev. 2010, 110, 2839. 20104890 10.1021/cr900236h

[12] Y. Shen , A. J. Shuhendler , D. Ye , J.‐J. Xu , H.‐Y. Chen , Chem. Soc. Rev. 2016, 45, 6725. 27711672 10.1039/c6cs00442c

[13] X. Zhao , J. Liu , J. Fan , H. Chao , X. Peng , Chem. Soc. Rev. 2021, 50, 4185. 33527104 10.1039/d0cs00173b

[14] M. Ethirajan , Y. Chen , P. Joshi , R. K. Pandey , Chem. Soc. Rev. 2011, 40, 340. 20694259 10.1039/b915149b

[15] H. Huang , B. Yu , P. Zhang , J. Huang , Y.u Chen , G. Gasser , L. Ji , H. Chao , Angew. Chem., Int. Ed. 2015, 54, 14255. 10.1002/anie.201507800 26447888

[16] C. Mari , V. Pierroz , S. Ferrari , G. Gasser , Chem. Sci. 2015, 6, 2660. 29308166 10.1039/c4sc03759f PMC5639435

[17] J. Li , Q.i Zhao , F. Shi , C. Liu , Y. Tang , Adv. Healthc. Mater. 2016, 5, 2967. 27925460 10.1002/adhm.201600868

[18] X. Li , D. Lee , J.‐D. Huang , J. Yoon , Angew. Chem., Int. Ed. 2018, 57, 10033.

[19] J. Li , K. Pu , Chem. Soc. Rev. 2019, 48, 38. 30387803 10.1039/c8cs00001h

[20] K. Qiu , Y. Chen , T. W. Rees , L. Ji , H. Chao , Coord. Chem. Rev. 2019, 378, 66.

[21] R. Zhang , Y. Xu , Y. Zhang , H. S. Kim , A. Sharma , J. Gao , G. Yang , J. S. Kim , Y. Sun , Chem. Sci. 2019, 10, 8348. 31803412 10.1039/c9sc03504d PMC6839587

[22] J. Li , Y. Liu , Y. Xu , L. Li , Y. Sun , W. Huang , Coord. Chem. Rev. 2020, 415.

[23] P.‐Y. Ho , S.‐Y. Lee , C. Kam , J. Zhu , G.‐G. Shan , Y. Hong , W.‐Y. Wong , S. Chen , Adv. Healthcare Mater. 2021, 10, e2100706. 10.1002/adhm.202100706 PMC11468684 34296536

[24] S. Tian , H. Bai , S. Li , Y. Xiao , X. Cui , X. Li , J. Tan , Z. Huang , D. Shen , W. Liu , P. Wang , B. Z. Tang , C.‐S. Lee , Angew. Chem., Int. Ed. 2021, 60, 11864. 10.1002/anie.202101406 33724623

[25] Z. Zhang , W. Xu , P. Xiao , M. Kang , D. Yan , H. Wen , N. Song , D. Wang , B. Z. Tang , ACS Nano 2021, 15, 10689. 34077187 10.1021/acsnano.1c03700

[26] M. Chiba , Y. Ichikawa , M. Kamiya , T. Komatsu , T. Ueno , K. Hanaoka , T. Nagano , N. Lange , Y. Urano , Angew. Chem., Int. Ed. 2017, 56, 10554. 10.1002/anie.201704793 28639393

[27] J. Jiang , Y. Qian , Z. Xu , Z. Lv , P. Tao , M. Xie , S. Liu , W. Huang , Q. Zhao , Chem. Sci. 2019, 10, 5085. 31183060 10.1039/c8sc05501g PMC6524665

[28] W. Sun , Y. Jian , M. Zhou , Y. Yao , N. Tian , C. Li , J. Chen , X. Wang , Q. Zhou , J. Med.Chem. 2021, 64, 7359. 34032114 10.1021/acs.jmedchem.0c02257

[29] S. Xu , Y. Yuan , X. Cai , C.‐J. Zhang , F. Hu , J. Liang , G. Zhang , D. Zhang , B. Liu , Chem. Sci. 2015, 6, 5824. 28791088 10.1039/c5sc01733e PMC5520955

[30] J. Qian , B. Z. Tang , Chem 2017, 3, 56.

[31] D. Wang , H. Su , R. T. K. Kwok , X. Hu , H. Zou , Q. Luo , M. M. S. Lee , W. Xu , J. W. Y. Lam , B. Z. Tang , Chem. Sci. 2018, 9, 3685. 29780499 10.1039/c7sc04963c PMC5935061

[32] R. Zhang , Y. Duan , B. Liu , Nanoscale 2019, 11, 19241. 31544188 10.1039/c9nr06012j

[33] J. Dai , X. Wu , S. Ding , X. Lou , F. Xia , S. Wang , Y. Hong , J. Med. Chem. 2020, 63, 1996. 32039596 10.1021/acs.jmedchem.9b02014

[34] Z. Zhao , H. Zhang , J. W. Y. Lam , B. Z. Tang , Angew. Chem. Int. Ed. 2020, 59, 9972. 10.1002/anie.201916729 32048428

[35] Y. Wang , S. Xu , L. Shi , C. Teh , G. Qi , B. Liu , Angew. Chem., Int. Ed. 2021, 60, 15072. 10.1002/anie.202017350 33887096

[36] X. Liu , C. Zhu , B. Z. Tang , Acc. Chem. Res. 2022, 55, 197. 34985255 10.1021/acs.accounts.1c00630

[37] Z. Zhou , J. Liu , T. W. Rees , H. Wang , X. Li , H. Chao , P. J. Stang , Proc. Natl. Acad. Sci 2018, 115, 5664. 29760069 10.1073/pnas.1802012115 PMC5984529

[38] Z. Zhou , J. Liu , J. Huang , T. W. Rees , Y. Wang , H. Wang , X. Li , H. Chao , P. J. Stang , Proc. Natl. Acad. Sci 2019, 116, 20296. 31548389 10.1073/pnas.1912549116 PMC6789806

[39] S. Gao , X. Yan , G. Xie , M. Zhu , X. Ju , P. J. Stang , Y.e Tian , Z. Niu , Proc. Natl. Acad. Sci 2019, 116, 23437. 31685638 10.1073/pnas.1911869116 PMC6876234

[40] H. Wang , C.‐H. Liu , K. Wang , M. Wang , H. Yu , S. Kandapal , R. Brzozowski , B. Xu , M. Wang , S. Lu , X.‐Q. Hao , P. Eswara , M.‐P. Nieh , J. Cai , X. Li , J. Am. Chem. Soc. 2019, 141, 16108. 31509694 10.1021/jacs.9b08484 PMC6849473

[41] S. Bhattacharyya , M. Venkateswarulu , J. Sahoo , E. Zangrando , M. De , P. S. Mukherjee , Inorg. Chem 2020, 59, 12690. 32806011 10.1021/acs.inorgchem.0c01777

[42] Y. Xu , W. Tuo , L. Yang , Y. Sun , C. Li , X. Chen , W. Yang , G. Yang , P. J. Stang , Y. Sun , Proc. Natl. Acad. Sci 2022, 61, e202110048.

[43] Q. Hu , M. Gao , G. Feng , B. Liu , Natl. Acad. Sci. 2014, 53, 14449.

[44] C.‐J. Zhang , Q. Hu , G. Feng , R. Zhang , Y. Yuan , X. Lu , B. Liu , Chem. Sci. 2015, 6, 4580. 28717475 10.1039/c5sc00826c PMC5500860

[45] B. Wang , M. Wang , A. Mikhailovsky , S. Wang , G. C. Bazan , Proc. Natl. Acad. Sci 2017, 56, 5113.

[46] H. Chen , S. Li , M. Wu , Z. H. Kenry , C. S. Lee , B. Liu , Proc. Natl. Acad. Sci. 2020, 59, 642.

[47] B. Rosenberg , L. Van Camp , T. Krigas , Nature 1965, 205, 698. 14287410 10.1038/205698a0

[48] N. Muhammad , Z. Guo , Curr. Opin. Chem. Biol. 2014, 19, 144. 24608084 10.1016/j.cbpa.2014.02.003

[49] H. Chong , E. Fron , Z. Liu , S. Boodts , J. Thomas , J. N. Harvey , J. Hofkens , W. Dehaen , M. Van Der Auweraer , M. Smet , Eur. J. Chem. 2017, 23, 4687. 10.1002/chem.201700015 28134471

[50] V. A. Semenov , Y. Y. Rusakov , D. O. Samultsev , L. B. Krivdin , Mendeleev Commun. 2019, 29, 315.

[51] M. Xuan , J. Shao , J. Zhao , Q.i Li , L. Dai , J. Li , Angew. Chem. 2018, 130, 6157. 10.1002/anie.201712996 29480962

[52] B. Hao , J. Wang , C. Wang , K.e Xue , M. Xiao , S. Lv , C. Zhu , Chem. Sci. 2022, 13, 4139. 35440990 10.1039/d2sc00381c PMC8985587

[53] M. J. Kasha , Discuss. Faraday Soc. 1950, 9, 14.

[54] C. M. Marian , WIRES. Comput. Mol. Sci. 2012, 2, 187;

[55] T. Wu , A. C. McCandlish , L. S. Gronenberg , S.‐S. Chng , T. J. Silhavy , D. Kahne , Proc. Natl. Acad. Sci. 2006, 103, 11754. 16861298 10.1073/pnas.0604744103 PMC1544242

[56] A. B. Nepomnyashchii , M. Bröring , J. Ahrens , A. J. Bard , J. Am. Chem. Soc. 2011, 133, 8633. 21563824 10.1021/ja2010219

