# Supplementary material for: Organo‐Ptii Complexes for Potent Photodynamic Inactivation of Multi‐Drug Resistant Bacteria and the Influence of Configuration
Source: Adv Sci (Weinh). 2024 Jan 31;11(14):2306936. doi: 10.1002/advs.202306936 (PMC11005693; doi:10.1002/advs.202306936)
Supplement: Supplementary file 1 — Supporting Information [file ADVS-11-2306936-s001.pdf]

ADVANCED SCIENCE
Open Access

Supporting Information

for *Adv. Sci.*, DOI 10.1002/advs.202306936

Organo-Pt^{II} Complexes for Potent Photodynamic Inactivation of Multi-Drug Resistant Bacteria and the Influence of Configuration

Hui Chong, Xuanwei Liu, Siyu Fang, Xiaofei Yang, Yuefei Zhang, Tianyi Wang, Lin Liu, Yinshi Kan, Yueqi Zhao, Hongying Fan, Jingqi Zhang, Xiaoyu Wang, Hang Yao, Yi Yang, Yijian Gao, Qi Zhao, Shengliang Li, Martin Plymoth, Juqun Xi, Yu Zhang, Chengyin Wang* and Huan Pang**

Supporting Information

Organo-Pt^{II} complexes for Potent Photodynamic Bacterial Inactivation and Influence of Configuration

Hui Chong, Xuanwei Liu, Siyu Fang, Xiaofei Yang, Yuefei Zhang, Tianyi Wang, Lin Liu, Yinshi Kan, Yueqi Zhao, Hongying Fan, Jingqi Zhang, Xiaoyu Wang, Hang Yao, Yi Yang, Yijian Gao, Qi Zhao, Shengliang Li, Martin Plymoth, Juqun Xi, Yu Zhang, Chengyin Wang* and Huan Pang**

Experimental Procedures

Determination of fluorescent quantum yield: The fluorescent quantum yield of ***cis*-BBP** and ***trans*-BBP** in water was determined using aqueous solution of Rhodamine B (96%) as reference.

DFT calculation: Density-Functional-Theory (DFT) calculations for structure optimization and electronic description of ***cis*-BBP** and ***trans*-BBP** was conducted with Gaussian 16 software package using B3LYP-D3/Def2-SVP/SDD method.[1]

$^1O_2$ detection: Rose Bengal (RB), ***cis*-BBP** and ***trans*-BBP** was dissolved in Milli-Q water to reach a final concentration of 2.50 μM. $^1O_2$ sensor 9,10-Anthracenediyl-bis(methylene)dimalonic acid (ABDA) was then added to these solutions with the final concentration of 50 μM. The resulting solution was then subjected to white light irradiation (100 mW/cm^2) for total 5 min. UV-vis spectra was recorded every min, and the absorbance decrease at 378 nm was used to characterized decomposition of ABDA by $^1O_2$.

Preparation of bacterial dispersion: A single colony of MDR-*A. baumannii* (Gram-negative) and *S. aureus* (Gram-positive) was cultured in 5 mL of liquid Luria-Broth (LB) culture medium at 37 °C for 3 hours. The bacterial were harvested by a centrifuge. The collected bacteria were then washed using 1×PBS for three times followed by resuspended in 1×PBS. The bacterial dispersion was adjusted to be 0.5 at 600 nm as measured by a UV-vis spectrometer.

Isothermal Titration Microcalorimetry (ITC): The calorimetric measurements were conducted using a Malvern MicroCalITC200 apparatus. The 200 μL of bacteria solution (MDR-*A. baumannii* and MRSA with OD_{600nm} = 0.5) was loaded into the sample cell. ***cis*-BBP** and ***trans*-BBP** solutions (0.01 mg/mL, 20 μL) were continuously titrated into the sample cell at 650 rpm stirring. The dilution heat of different samples has been subtracted during the analysis. All of the measurements were conducted at 25.00 ± 0.01 °C. The accuracy of the calorimeter was periodically calibrated electrically and verified by measuring the dilution enthalpy of concentrated sucrose solution. The experiments were repeated at least twice with deviation within ±

4%. The Gibbs free energy (ΔG_b) was calculated from $\Delta G_b = -RT\ln K$ and the entropy change (ΔS_b) from $\Delta S_b = (\Delta H_b - \Delta G_b)/T$.

Confocal laser scanning microscopy (CLSM): 100 μL of MDR *A. baumannii* dispersion (final concentration of 1.0×10^7 CFU/mL) was mixed with 100 μL of DAPI and 100 μL of Pt^{II} PSs aqueous solution. The resulting mixture was subsequently incubated for 20 min in dark. Then, the bacteria were collected by centrifuge and dispersed with 100 μL of 1×PBS and distributed in quartz slide for fluorescent imaging. The excitation wavelength for DAPI and PSs was 405 and 488 nm, respectively. The detection wavelength range for DAPI and PSs was 410-491 and 493-598 nm.

***In vitro* inactivation of bacteria**: 20 μL of bacteria dispersion (MDR- *A. baumannii* and *S. aureus*) were transferred to a solid LB culture medium on 24-well plates (1.0 $\times10^7$ CFU/mL). PBS solution of organometallic PSs was added to bacteria dispersion. The final concentration of ***cis*-BBP** and ***cis*-BPP** for MDR- *A. baumannii* was 0.05, 0.1, 0.2 and 0.4 μg/mL; for *S. aureus* was 0.05, 0.10, 0.15, 0.20 and 0.25 μg/mL. The final concentration of ***trans*-BBP** and ***trans*-BPP** for MDR- *A. baumannii* was 1.00, 1.50, 2.00 and 2.50 μg/mL; for *S. aureus* was 0.50, 1.00, 1.50, and 2.00 μg/mL. After incubation of bacteria with PSs for 20 min in dark, white light (100 mW/cm^2) was applied on the bacteria for 1, 2, 3, 4 and 5 min. The bacteria with addition of PSs was used as control. In the dark toxicity experiment, no light irradiation was applied on bacteria. After these treatments, 10 μL of bacteria dispersion was taken and diluted 10^3 folds and cultured on new solid LB medium in dark for 24 hours. All experiments were repeated for 3 times. The antibacterial efficiency (E_b) was calculated using the formula:

$$E_b = \left(\frac{C^0 - C}{C^0}\right) \times 100\% \qquad (1)$$

Where, C^0 stands for the colony number of control group, C stands for the colony number of individual experimental group. The antibacterial efficiency was also shown in form of logarithm of 10 after conversion in the figures.

***In vivo* inactivation of bacteria**: The in vivo experiments were authorized by the Science and Technology Department of Jiangsu Province and conducted with the approval of the Medical Ethics Committee of Yangzhou University Medical Academy

(YXYLL-2022-44). Methicillin resistant *S. aureus* (MRSA) infected mice were randomly divided into six groups (n = 6), namely Dark control (0.9% NaCl), Light control (0.9% NaCl and light irradiation of 30 J/cm^2), ***cis*-BBP** dark (50 μL of 0.10 μg/mL ***cis*-BBP** in dark), ***cis*-BBP** light (50 μL of 0.10 μg/mL ***cis*-BBP** and light irradiation), ***trans*-BBP** dark (50 μL of 0.10 μg/mL ***trans*-BBP** in dark) and ***trans*-BBP** light (50 μL of 0.10 μg/mL ***trans*-BBP** and light irradiation). The PS dispersion was uniformly smeared on the infected wound for successive three days and followed by application of light irradiation. The area of wound of different experimental groups was calculated and the photos of mice models were taken on day 0, 1, 3, 5, 7 and 14. The mice were sacrificed on 14th day, and vital organs and blood were collected for biosafety analysis. The relative wound area was calculated according to the following formula:

$$\text{Relative wound area} = \left(\frac{S_n}{S_0}\right) \times 100\% \qquad (2)$$

Where, S_n was wound area on certain day after treatment in different experimental groups. S_0 was wound area on day 0 in different experimental groups.

Hematoxylin-Eosin staining: The collected wound tissue from different experimental groups on day 7 was first dehydrated with treatment of 80% and 90% ethanol for 40 min and 95% and 100% ethanol for 20 min, respectively. Next, dehydrated samples were fixed with 4% paraformaldehyde at room temperature for 24 hours. Then the samples were embedded in paraffin and subsequently cut into 6 μm slides. Finally, the samples were stained with hematoxylin and eosin and the corresponding photos were taken.

Biosafety evaluation: After 14 days, the mice were sacrificed, and the blood was drawn for routine serum biochemical and hematological examination. The tissues of the heart, liver, spleen, lung, and kidney were collected to observe the in vivo toxicity of different Pt^{II} complexes to major organs. After the tissues were collected, they were washed with normal saline, fixed with 10% formalin, embedded with OCT, and cut into 6μm thick tissue slices. Staining was performed according to the H&E staining kit scheme. Observe and photograph under an optical microscope.

Immunofluorescence: Each group's collected wounded skin tissues were fixed with

10% formalin, embedded by OCT, and cut into 6 μm thick tissue slices. Slices were incubated with 0.25% Triton PBS solution for 25 minutes at room temperature and washed 3 times with PBS for 5 minutes/time. Goat serum prepared in 10% PBS was blocked at room temperature for 30 minutes, and washed 3 times with PBS, 5 minutes each time. Antibody CD68 (rabbit, Proteintech, 1:200), antibody CD163 (rabbit, Proteintech, 1:200) and VEGF (rabbit, Proteintech, 1:200) were diluted in 10% goat serum. The negative control group was incubated with 10% goat serum. Incubated overnight at 4 °C in a humid chamber, then washed with PBS 3 times for 5 min each. Secondary antibodies goat anti-rabbit IgG labeled by Alexa 488 (Abcam, 1:500) and goat anti-rabbit IgG labeled by Alexa 568 (Abcam, 1:500) were diluted in PBS. Negative controls were incubated with mixed secondary antibodies, incubated for 2 h at 37 °C, and then washed with PBS 3 times for 10 min each. Nuclei were stained with DAPI for 30 minutes and washed 3 times with PBS for 5 minutes each. Fluorescence was observed by fluorescence inverted microscope after the tablets were sealed with a fluorescent sealing agent. The relatively expression of individual inflammatory related factors was characterized by comparing the fluorescent intensity of DAPI and certain stained protein in each experimental group.

Cell culture: L929 cells were cultured in DMEM medium supplemented with 10% of FBS. The cells were harvested through centrifugation and cultured at 37 °C in 5% CO_2 atmosphere.

Cell viability: L929 cells were seeded in 96-well flat-bottom plats at a density of 10^4 cells/well. The cells were then incubated with PSs with same final concentrations as in antibacterial experiments for 24 hours in dark. For light experiments, the cells were irradiated with white light (100 mW/cm^2) for 1-5 min. For dark experiments, no light irradiation was applied. For two experiments, cells without addition of PSs were used as control. Subsequently, culture medium was replaced by fresh DMEM containing MTT (0.5 mg/mL). The cells were incubated for another 4 hours and 100 μL of DMSO was added into each well. The absorption values of each well at 570 nm was recorded. All experiments were repeated for 3 times.

Reference

[1] a) F. Weigend, *Phys. Chem. Chem. Phys.* **2006**, *8*, 1057-1065; b) F. Weigend, R. Ahlrichs, *Phys. Chem. Chem. Phys.* **2005**, *7*, 3297-3305; c) P. Schwerdtfeger, M. Dolg, W. H. E. Schwarz, G. A. Bowmaker, P. D. W. Boyd, *J. Chem. Phys.* **1989**, *91*, 1762-1774; d) M. Dolg, U. Wedig, H. Stoll, H. Preuss, *J. Chem. Phys.* **1987**, *86*, 866-872; e) M. J. Frisch, G. W. Trucks, H. B. Schlegel, G. E. Scuseria, M. A. Robb, J. R. Cheeseman, G. Scalmani, V. Barone, G. A. Petersson, H. Nakatsuji, X. Li, M. Caricato, A. V. Marenich, J. Bloino, B. G. Janesko, R. Gomperts, B. Mennucci, H. P. Hratchian, J. V. Ortiz, A. F. Izmaylov, J. L. Sonnenberg, Williams, F. Ding, F. Lipparini, F. Egidi, J. Goings, B. Peng, A. Petrone, T. Henderson, D. Ranasinghe, V. G. Zakrzewski, J. Gao, N. Rega, G. Zheng, W. Liang, M. Hada, M. Ehara, K. Toyota, R. Fukuda, J. Hasegawa, M. Ishida, T. Nakajima, Y. Honda, O. Kitao, H. Nakai, T. Vreven, K. Throssell, J. A. Montgomery Jr., J. E. Peralta, F. Ogliaro, M. J. Bearpark, J. J. Heyd, E. N. Brothers, K. N. Kudin, V. N. Staroverov, T. A. Keith, R. Kobayashi, J. Normand, K. Raghavachari, A. P. Rendell, J. C. Burant, S. S. Iyengar, J. Tomasi, M. Cossi, J. M. Millam, M. Klene, C. Adamo, R. Cammi, J. W. Ochterski, R. L. Martin, K. Morokuma, O. Farkas, J. B. Foresman, D. J. Fox, Guassian 16, Rev. C.01, Wallingford, CT, **2016**.

Table S1. Oscillator factor (*f*), vertical excited energies (eV) and wavelength (nm) and for ***cis*-BBP**

S_n	f	Excited Energy (eV)	Wavelength (nm)	T_n	Excited Energy (eV)
S_1	0.0016	1.3795	898.76	T_1	1.3095
S_2	0.0004	1.5861	781.67	T_2	1.3805
S_3	0.0018	1.6654	744.46	T_3	1.4706
S_4	0.0051	1.7140	723.38	T_4	1.5895
S_5	0.0006	1.7547	706.59	T_5	1.6533
S_6	0.0000	2.0269	611.70	T_6	1.6946
S_7	0.0125	2.2343	554.92	T_7	1.7481
S_8	0.0022	2.2886	541.76	T_8	2.0259
S_9	0.0093	2.3776	521.47	T_9	2.2035
S_{10}	0.0334	2.4085	514.77	T_{10}	2.2794

Table S2. Oscillator factor (*f*), vertical excited energies (eV) and wavelength (nm) and for ***trans*-BBP**

S_n	*f*	Excited Energy (eV)	Wavelength (nm)	T_n	Excited Energy (eV)
S_1	0.0028	1.5143	818.73	T_1	1.3873
S_2	0.0014	1.5178	816.87	T_2	1.3879
S_3	0.0161	1.5636	792.93	T_3	1.5252
S_4	0.0046	1.5723	788.57	T_4	1.5283
S_5	0.0003	1.9267	641.99	T_5	1.5442
S_6	0.0003	1.9313	540.87	T_6	1.5480
S_7	0.1146	2.2923	537.78	T_7	1.9230
S_8	0.0018	2.3055	485.19	T_8	1.9273
S_9	0.0001	2.5554	484.32	T_9	2.2012
S_{10}	0.0002	2.5600	641.99	T_{10}	2.2041

Table S3. Spin orbit coupling constants (ξ, cm^{-1}) between the lowest singlet exited state (S_1) and different triplet states for ***cis*-BBP** and ***trans*-BBP**.

***cis*-BBP**	S_1-T_1	60.66836902
	S_1-T_2	9.033238622
	S_1-T_3	27.47137783
***trans*-BBP**	S_1-T_1	4.660407707
	S_1-T_2	3.675567983
	S_1-T_3	15.92091078
	S_1-T_4	18.99091888

Table S4. ζ Potential of MDR *A. baumannii* and MRSA treated with 2.00 μg/mL of ***cis*-BBP** and ***trans*-BBP**

**Bacteria**	**No Treatment**	***cis*-BBP**	***trans*-BBP**
MDR *A. baumannii*	-14.97 ± 1.68 mV	-1.69 ± 0.65 mV	-9.32 ± 0.67 mV
S. aureus	-15.70 ± 2.07 mV	-3.63 ± 1.88 mV	-8.36 ± 0.55 mV

Table S5. Binding constant (K), number of binding sites (N), ΔH_b, ΔS_b and ΔH_b estimated from the ITC fitting curve.

	***cis*-BBP** on MDR *A. baumannii*	***trans*-BBP** on MDR *A. baumannii*	***cis*-BBP** on MRSA	***trans*-BBP** on MRSA
N	$7.93 \pm 0.08 \times 10^5$	$6.10 \pm 0.06 \times 10^5$	$7.15 \pm 0.07 \times 10^5$	$9.87 \pm 0.07 \times 10^5$
K (M^{-1})	$1.69 \pm 0.22 \times 10^6$	$1.30 \pm 0.13 \times 10^6$	$1.36 \pm 0.15 \times 10^6$	$7.64 \pm 0.92 \times 10^5$
ΔH_b (kcal/mol)	-3.89±0.055	-0.98±0.013	-3.17±0.044	-1.76±0.035
ΔS_b (cal/mol/deg)	15.4	24.7	17.4	21.0
$T\Delta S_b$ (kcal/mol)	4.59	7.36	5.19	6.26
ΔG_b (kcal/mol)	-8.49	-8.35	-8.01	-8.36

Table S6. Anti-MDR *A. baumannii* efficiency of ***cis*-BBP** in dark under different incubation time.

Incubation Time (min)	Anti-MDR *A. baumannii* Efficiency (%)			
	Concentration (μg/mL)			
	**0.05**	**0.10**	**0.20**	**0.40**
1	19.15±0.10	12.61±0.07	13.29±0.06	10.99±0.10
2	26.22±0.12	16.70±0.06	22.72±0.01	17.65±0.06
3	33.06±0.14	19.72±0.07	32.57±0.07	31.74±0.05
4	34.34±0.06	30.80±0.03	44.64±0.08	43.20±0.02
5	40.98±0.04	35.60±0.05	45.86±0.03	47.98±0.04

Table S7. Anti-MDR *A. baumannii* efficiency of ***trans*-BBP** in dark under different incubation time

Incubation Time (min)	Anti-MDR *A. baumannii* Efficiency (%)			
	Concentration (μg/mL)			
	**1.00**	**1.50**	**2.00**	**2.50**
1	19.18±0.04	18.30±0.04	27.81±0.07	31.00±0.05
2	30.75±0.05	27.99±0.04	35.09±0.08	46.59±0.04
3	40.32±0.05	33.24±0.07	41.67±0.04	53.58±0.05
4	42.56±0.03	39.48±0.06	44.75±0.04	57.33±0.04
5	49.35±0.02	48.14±0.06	53.31±0.02	59.51±0.02

Table S8. Anti-MDR *A. baumannii* efficiency of ***cis*-BBP** under different light irradiation dose.

Light irradiation dose (J/cm^2)	Anti-MDR *A. baumannii* Efficiency (%)			
	Concentration (μg/mL)			
	**0.05**	**0.10**	**0.20**	**0.40**
6	30.50±0.09	63.44±0.06	92.91±0.03	99.58±0.00[a]
12	46.54±0.04	92.29±0.01	97.09±0.01	99.94±0.00[a]
18	44.34±0.01	94.50±0.01	98.36±0.02	99.97±0.00[a]
24	59.79±0.07	96.35±0.01	99.40±0.00	99.99±0.00[a]
30	58.01±0.10	97.79±0.01	99.63±0.00	99.99±0.00[a]

a: Error below 0.01.

Table S9. Anti-MDR *A. baumannii* efficiency of ***trans*-BBP** under different light irradiation dose.

Light irradiation dose (J/cm^2)	Anti-MDR *A. baumannii* Efficiency (%)			
	Concentration (μg/mL)			
	**0.50**	**1.00**	**1.50**	**2.00**
6	35.95±0.08	44.12±0.03	65.62±0.04	74.96±0.10
12	54.84±0.05	46.76±0.02	80.81±0.03	89.19±0.04
18	60.64±0.10	78.87±0.03	88.49±0.02	93.62±0.01
24	75.53±0.06	80.68±0.10	94.93±0.01	97.48±0.01
30	82.80±0.02	92.53±0.01	97.33±0.00[a]	99.29±0.00[a]

a: Error below 0.01.

Table S10. Anti-MRSA efficiency of ***cis*-BBP** in dark under different incubation time.

Incubation Time (min)	Anti-MRSA Efficiency (%)				
	Concentration (μg/mL)				
	**0.05**	**0.10**	**0.15**	**0.20**	**0.25**
1	12.30±0.03	8.60±0.01	6.39±0.03	8.53±0.03	6.09±0.03
2	21.18±0.06	17.28±0.03	17.81±0.03	15.51±0.04	16.34±0.10
3	26.38±0.06	23.22±0.02	20.33±0.01	21.38±0.08	22.37±0.07
4	33.55±0.04	33.75±0.06	35.40±0.11	30.11±0.03	30.66±0.03
5	40.01±0.03	38.46±0.05	41.75±0.08	39.86±0.07	42.08±0.10

Table S11. Anti-MRSA efficiency of ***trans*-BBP** in dark under different incubation time.

Incubation Time (min)	Anti-MRSA Efficiency (%) Concentration (μg/mL)			
	**0. 50**	**1.00**	**1.50**	**2.00**
1	9.98±0.08	14.08±0.01	20.20±0.06	19.35±0.03
2	14.62±0.06	21.15±0.03	26.64±0.01	37.34±0.08
3	21.92±0.05	33.11±0.05	33.40±0.02	39.47±0.06
4	24.71±0.05	32.54±0.03	38.47±0.01	45.87±0.06
5	29.34±0.06	36.68±0.02	43.05±0.03	47.69±0.05

Table S12. Anti-MRSA efficiency of ***cis*-BBP** under different light irradiation dose.

Light irradiation dose (J/cm^2)	Anti-MRSA Efficiency (%)				
	Concentration (μg/mL)				
	**0.05**	**0.10**	**0.15**	**0.20**	**0.25**
6	81.43±0.21	98.51±0.02	99.87±0.00[a]	99.98±0.00[a]	99.97±0.00[a]
12	89.66±0.15	99.79±0.00[a]	99.99±0.00[a]	99.99±0.00[a]	99.99±0.00[a]
18	95.34±0.06	99.95±0.00[a]	99.99±0.00[a]	99.99±0.00[a]	99.99±0.00[a]
24	97.96±0.02	99.97±0.00[a]	99.99±0.00[a]	99.99±0.00[a]	100.00±0.00[a]
30	99.30±0.00[a]	99.97±0.00[a]	99.99±0.00[a]	99.99±0.00[a]	100.00±0.10

a: Error below 0.01.

Table S13. Anti-MRSA efficiency of ***trans*-BBP** under different light irradiation dose.

Light irradiation dose (J/cm^2)	Anti-MRSA Efficiency (%)			
	Concentration (μg/mL)			
	**0.50**	**1.00**	**1.50**	**2.00**
6	38.86±0.01	86.78±0.01	99.77±0.00[a]	99.99±0.00[a]
12	44.62±0.03	92.32±0.01	99.88±0.00[a]	99.99±0.00[a]
18	53.73±0.02	93.85±0.01	99.92±0.00[a]	99.99±0.00[a]
24	64.43±0.06	95.73±0.01	99.94±0.00[a]	100.00±0.00[a]
30	74.48±0.03	96.71±0.01	99.97±0.00[a]	100.00±0.00[a]

a: Error below 0.01.

Table S14. MIC_{50} ratio of ***trans*-BBP/*cis*-BBP** towards MDR *A. baumannii* and MRSA under different dose of light irradiation

Light dose (J/cm^2)	MIC_{50} ratio (***trans*-BBP/*cis*-BBP**)	
	MDR *A. baumannii*	MRSA
6	16.84	21.29
12	22.22	19.64
18	17.96	18.89
24	17.71	17.69
30	14.58	17.20

Table S15. MIC_{90} ratio of ***trans*-BBP/*cis*-BBP** towards MDR *A. baumannii* and MRSA under different dose of light irradiation

Light dose (J/cm^2)	MIC_{50} ratio (***trans*-BBP/*cis*-BBP**)	
	MDR *A. baumannii*	MRSA
6	16.13	22.36
12	24.02	19.22
18	19.27	19.58
24	19.10	19.13
30	15.00	18.67

Table S16. Wound remain ratio of MRSA infected ICR-mice under different treatments within 14 days.

Time (day)		0	1	3	5	7	14
Wound remain ratio (%)	**Dark Control**	100	71.96±3.39	63.88±4.32	51.72±2.23	37.98±0.91	21.53±4.36
	**Light Control**	100	66.89±6.58	46.69±6.48	41.22±4.91	28.81±6.57	16.69±2.14
	***trans*-BBP Dark**	100	70.97±5.33	47.06±6.55	41.65±5.09	34.33±6.97	16.32±1.63
	***trans*-BBP Light**	100	56.20±3.77	45.89±6.37	41.95±9.26	35.63±9.61	14.99±1.55
	***cis*-BBP Dark**	100	73.23±1.95	57.16±2.84	38.57±10.69	29.34±12.32	9.01±0.42
	***cis*-BBP Light**	100	60.65±7.60	45.43±2.43	36.03±3.70	23.87±11.61	5.27±0.44

Table S17. Comparision of ***cis*-BBP** with other organo-Pt^{II} PSs for antibacterial performance.

No.	Bacteria	Light Doses	Light type	Efficiency	Reference
1	*S. aureus*<br>*E. coli*	36 J/cm^2	450 nm, Laser	~70%, 2 μM<br>~30%, 2 μM	*Angew. Chem. Int. Ed*. **2021**, *60*, 2–8
2	*E. coli*	22.5 J/cm^2	420 nm	96.3%, 40 μM	*Proc. Natl. Acad. Sci*. **2019**, 47, 23437–23443.
3	*MRSA*<br>*P. aeruginosa*	40 W, 12 hours	White LED Bulb	MIC = 30 μM	*Inorg. Chem*. **2020**, 59, 12690−12699
4	*MDR A. baumannii*<br>MRSA	12 J/cm^2<br>18 J/cm^2	White Light	99.94%, 0.40 μM<br>99.95%, 0.10 μM	This Work

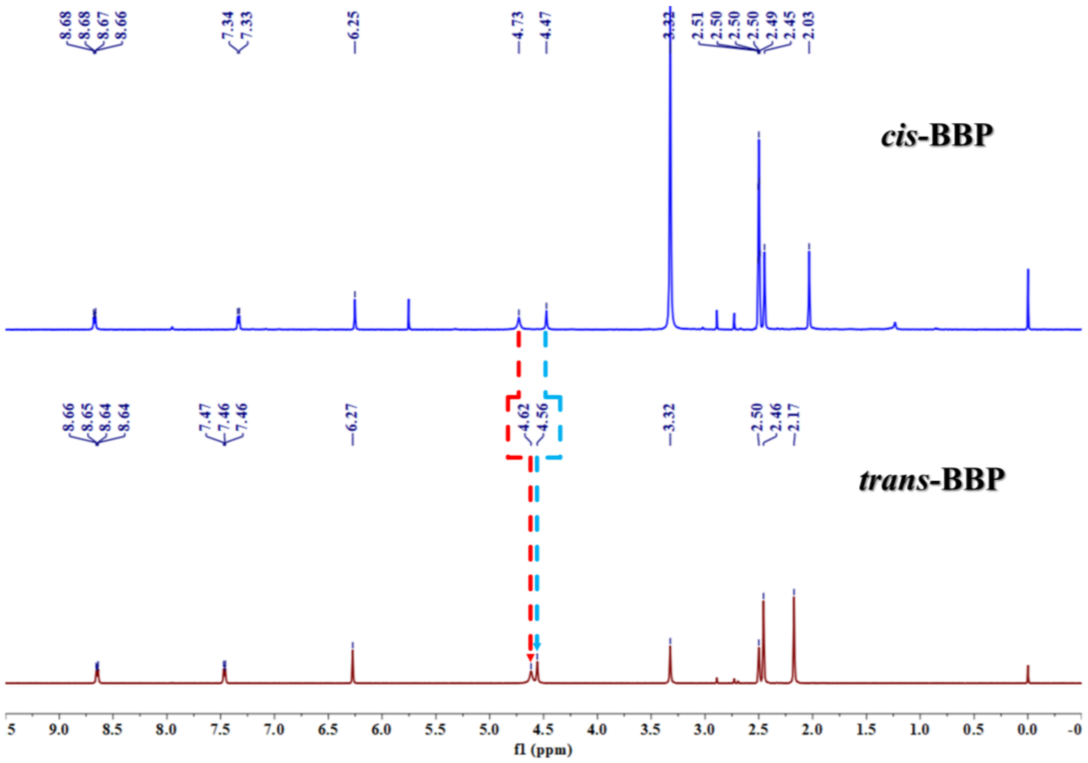

Figure S1. ^{1}H NMR comparison of ***cis*-BBP** and ***trans*-BBP** in DMSO-d_6, 300 MHz, 298 k.

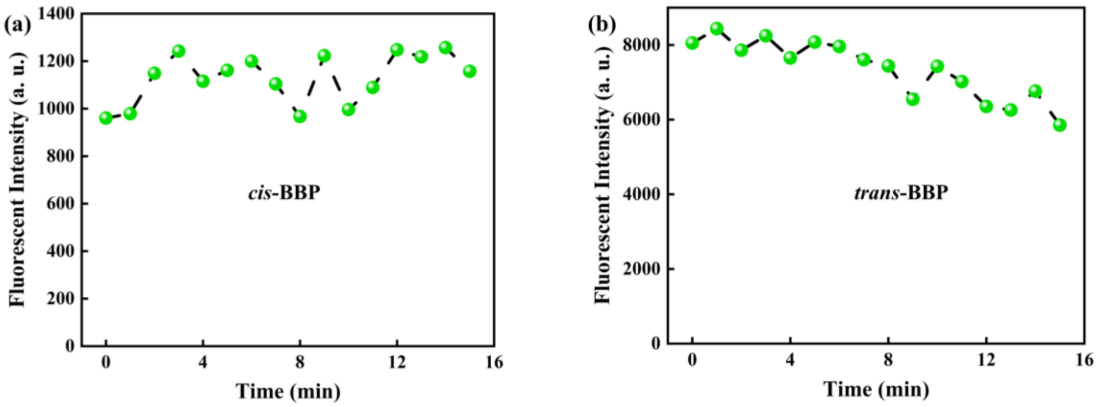

Figure S2. Photostability of ***cis*-BBP** and ***trans*-BBP**.

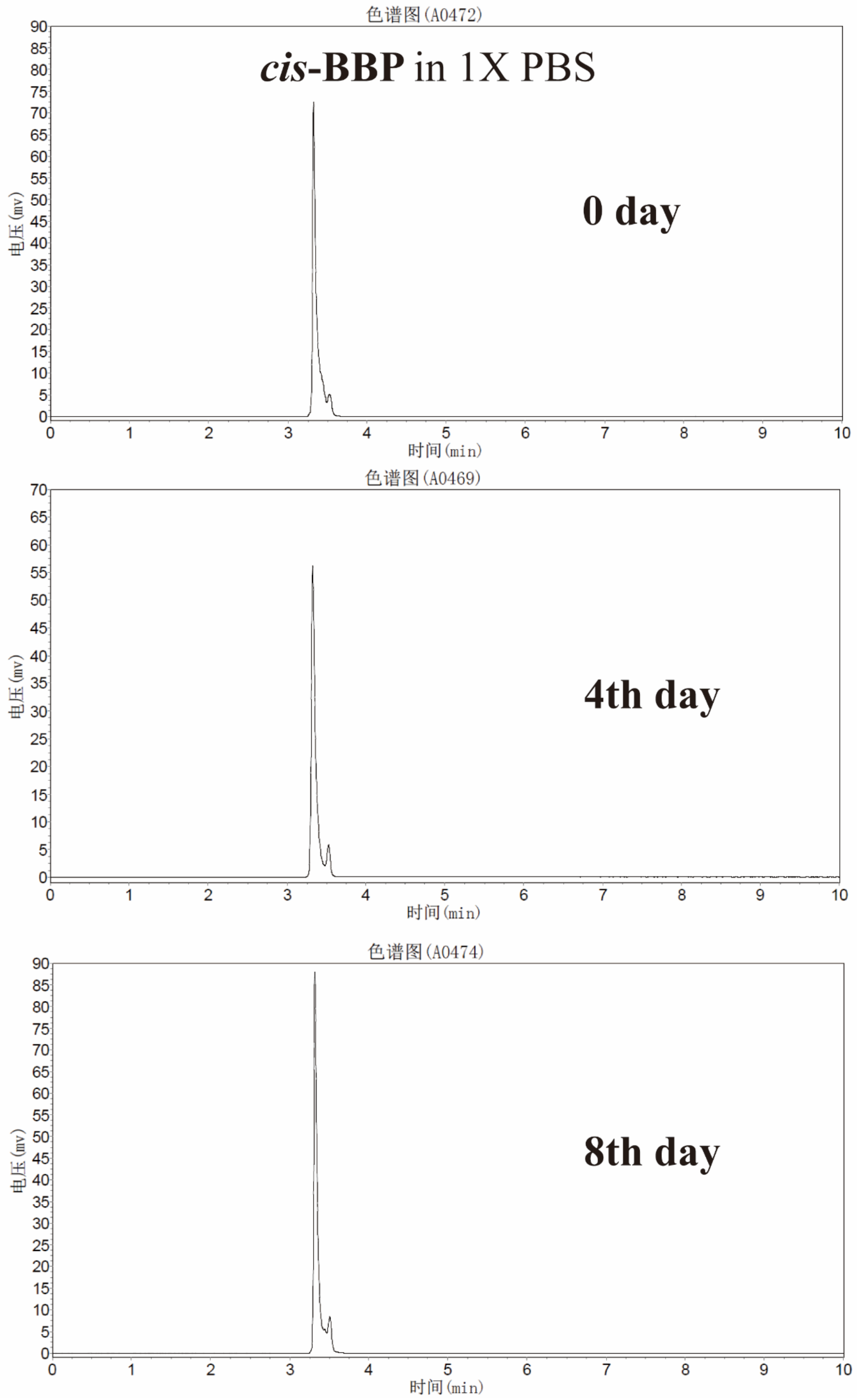

Figure S3. Stability of ***cis*-BBP** in 1X PBS at 37 °C for different days characterized by HPLC. [***cis*-BBP**] = 10 μg/mL.

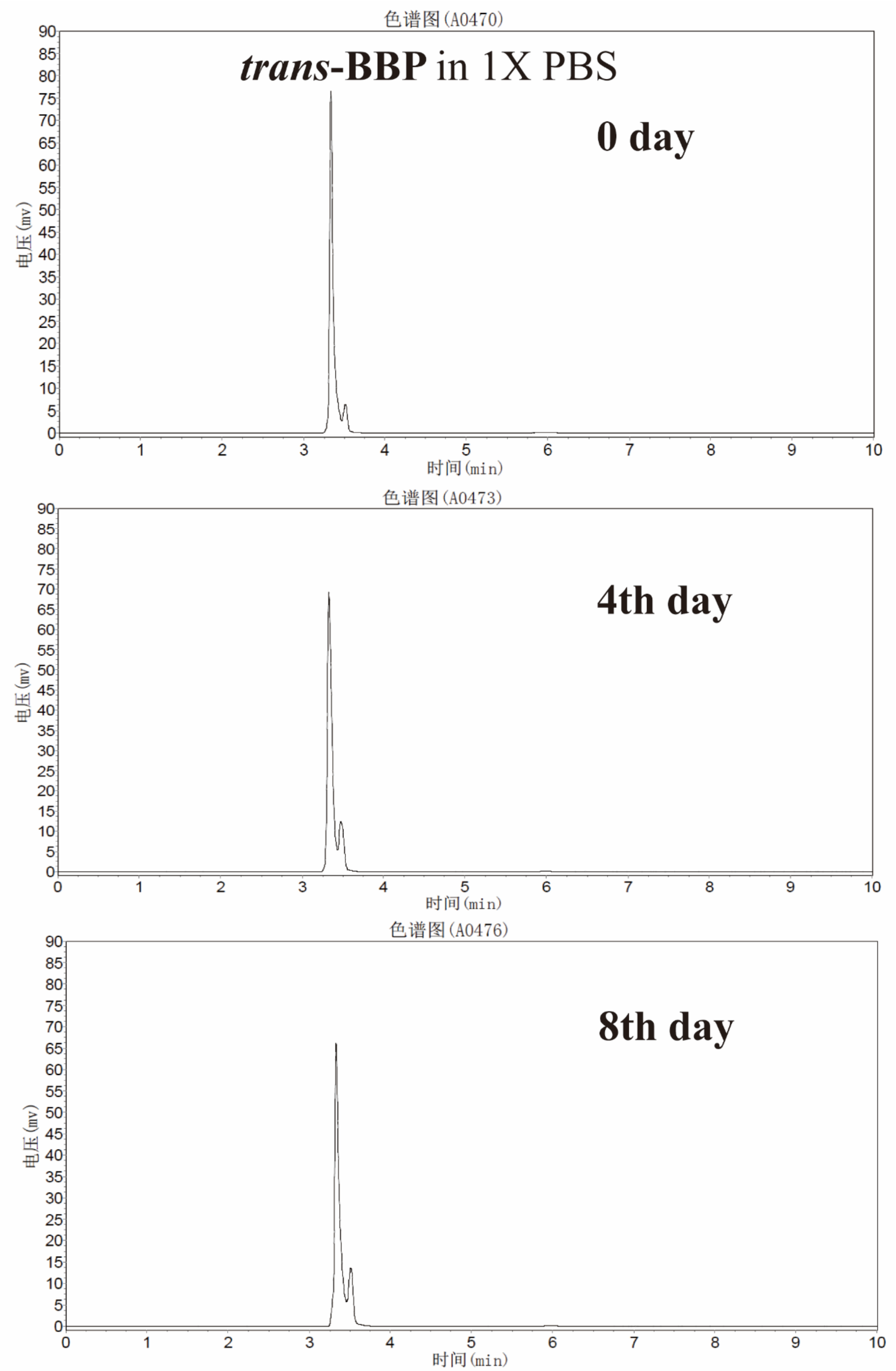

Figure S4. Stability of ***trans*-BBP** in 1X PBS at 37 ℃ for different days characterized by HPLC. [***trans*-BBP**] = 10 μg/mL.

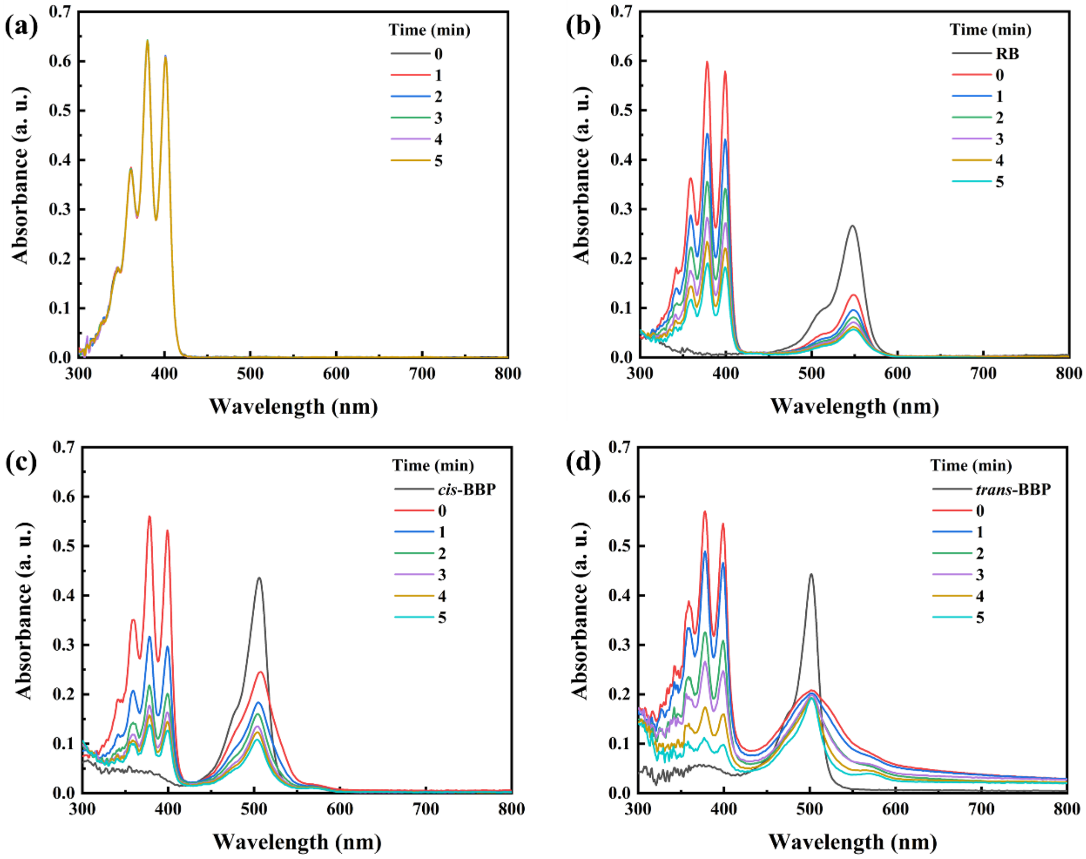

Figure S5. Singlet oxygen measurements under white light irradiation for ABDA (a), Rose Bengal (b), ***cis*-BBP** (c) and ***trans*-BBP** (d).

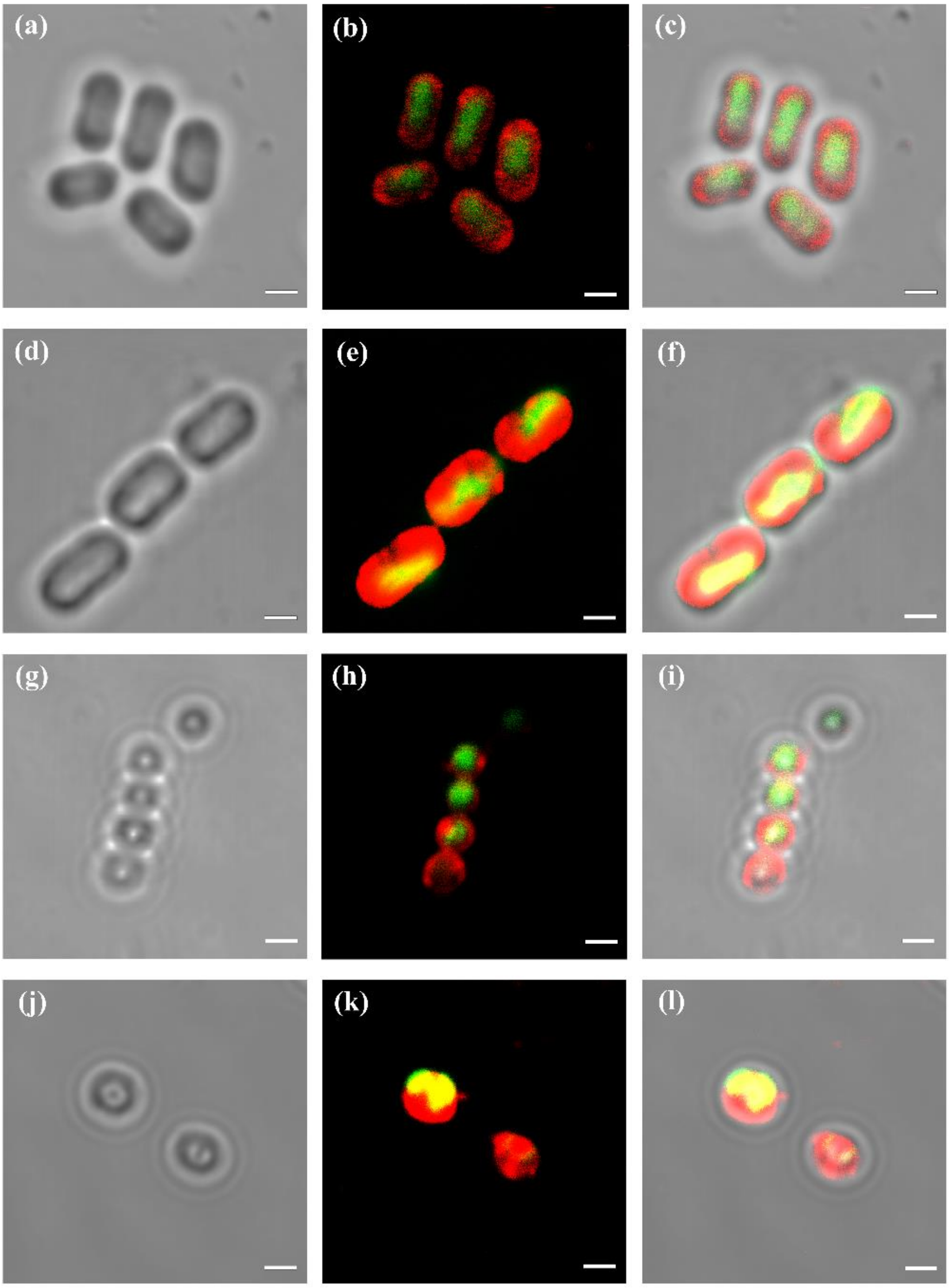

Figure S6. CLSM images of MDR *A. baumannii* and *S. aureus* stained with PSs and DAPI. (a) and (d) DIC images of MDR *A. baumannii*. (b) MDR *A. baumannii* stained with DAPI (green color) and ***cis*-BBP** (red color). (c) merge of (a) and (b). (e) MDR *A. baumannii* stained with DAPI (green color) and ***trans*-BBP** (red color). (f) merge of (d) and (e). (g) and (j) DIC images of MDR *S. aureus*. (h) MDR *S. aureus* stained with DAPI (green color) and ***cis*-BBP** (red color). (i) merge of (g) and (h). (k) MDR *S. aureus* stained with DAPI (green color) and ***cis*-BBP** (red color). (l) merge of (j) and (k). Excitation wavelengths for PSs and DAPI were 488 and 405 nm, respectively. [PSs] = 3.00 μg/mL, [DAPI] = 3.30 μg/mL, scale bar = 1 μm.

MDR *A. baumannii*

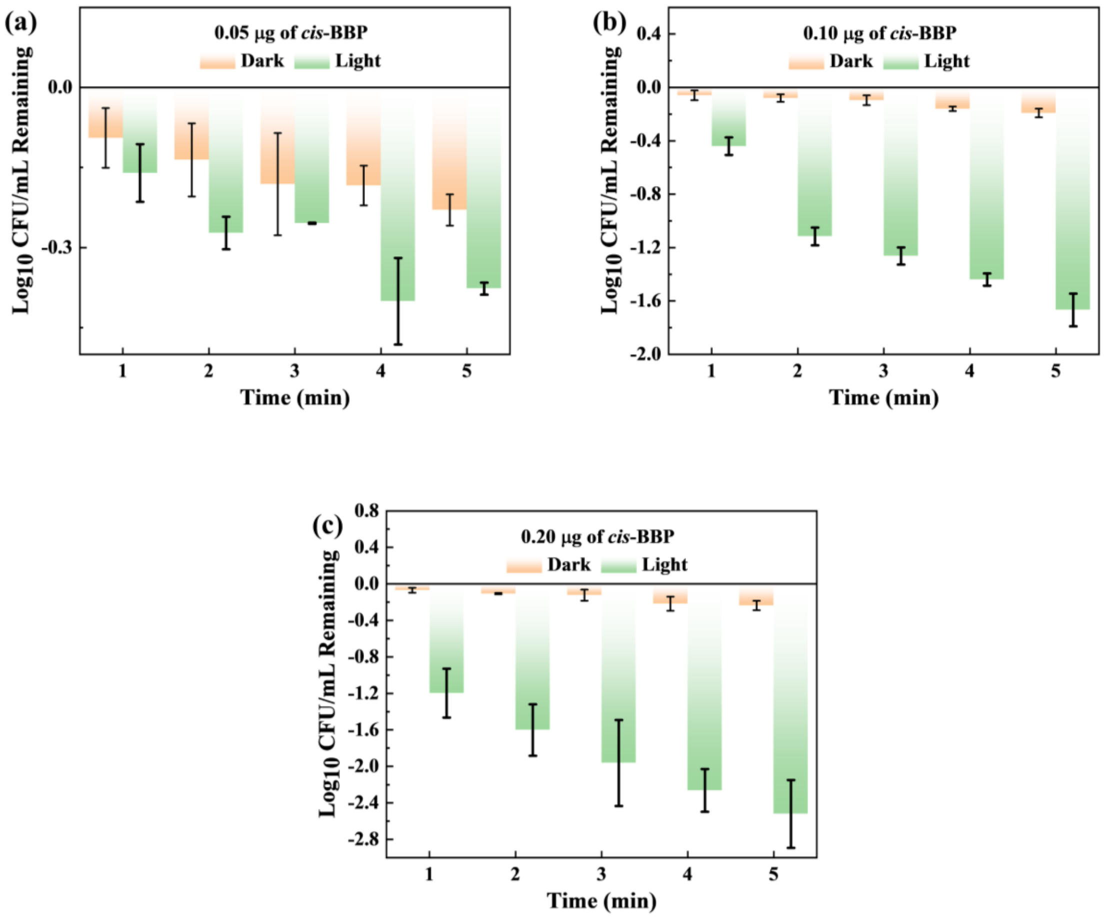

Figure S7. Anti-MDR *A. baumannii* performance of ***cis*-BBP** with concentration of 0.05 μg/mL (a), 0.10 μg/mL (b) and 0.20 μg/mL (c) in dark and under white light irradiation for 1-5 min.

MDR *A. baumannii*

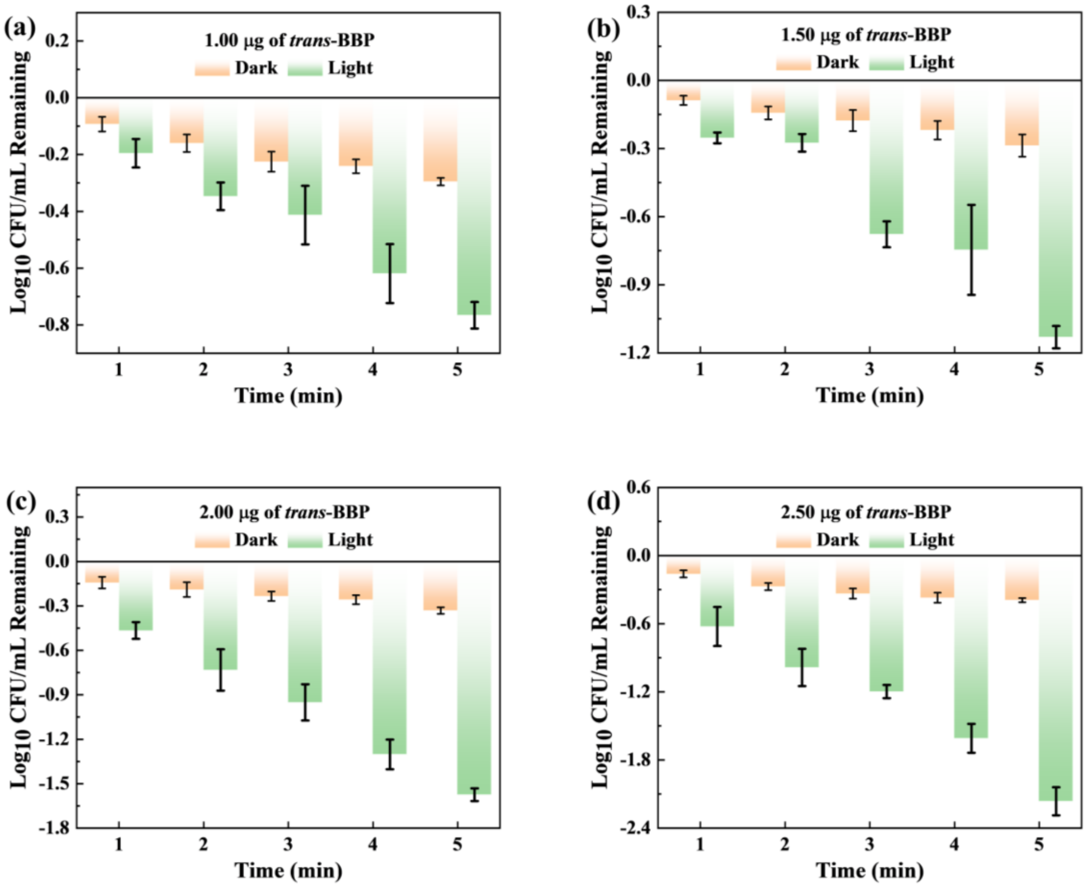

Figure S8. Anti-MDR *A. baumannii* performance of ***trans*-BBP** with concentration of 1.00 μg/mL (a), 1.50 μg/mL (b), 2.00 μg/mL (c) and 2.50 μg/mL (d) in dark and under white light irradiation for 1-5 min.

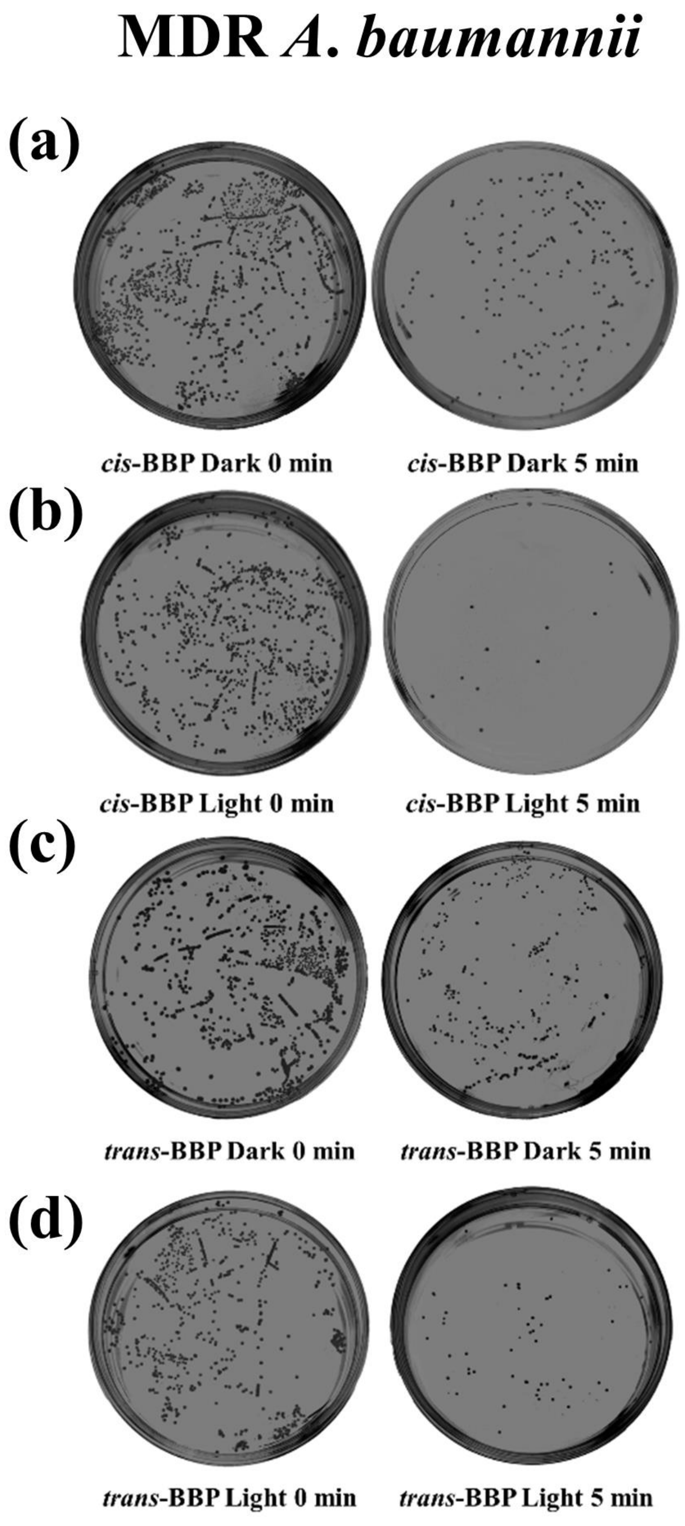

Figure S9. Photographs of MDR *A. baumannii* colonies with exposure to ***cis*-BBP** (a) in dark and under light irradiation (b). And ***trans*-BBP** (c) in dark and under light irradiation (d).

MDR *A. baumannii*

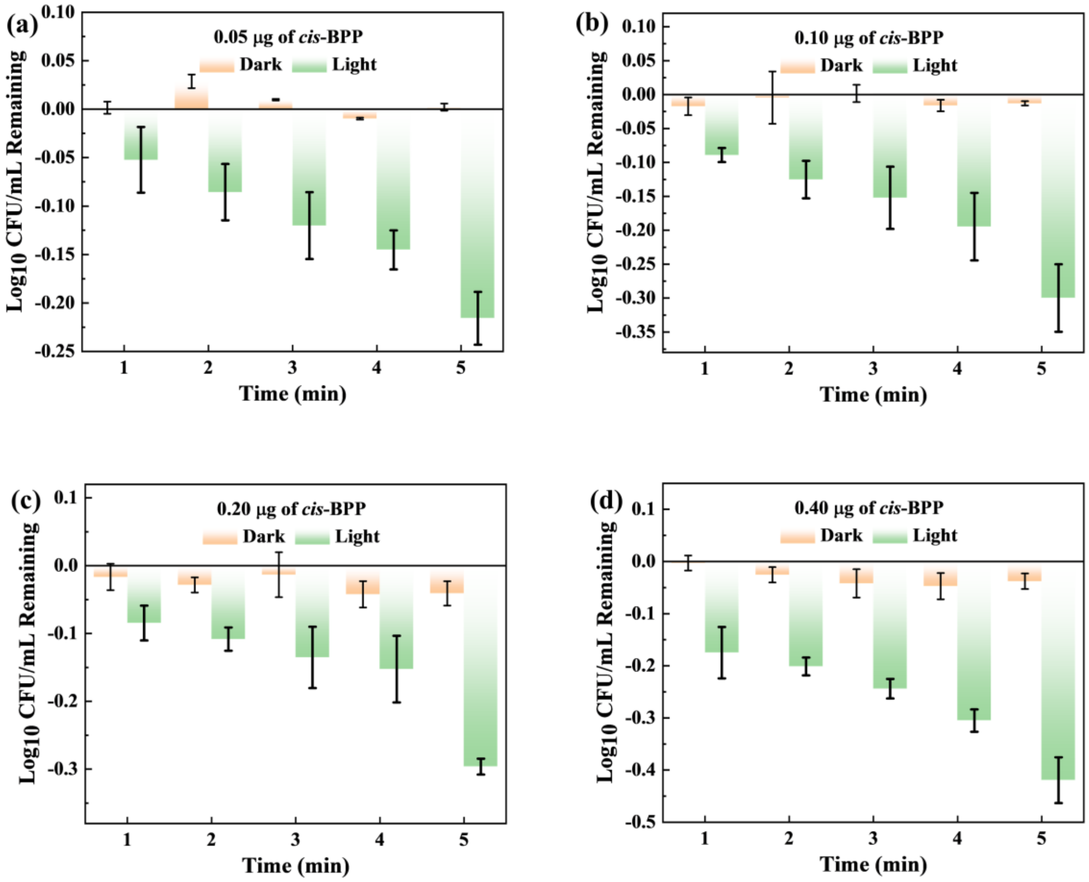

Figure S10. Anti-MDR *A. baumannii* performance of ***cis*-BPP** with concentration of 0.05 µg/mL (a), 0.10 µg/mL (b), 0.20 µg/mL (c) and 0.40 µg/mL (d) in dark and under white light irradiation for 1-5 min.

MDR *A. baumannii*

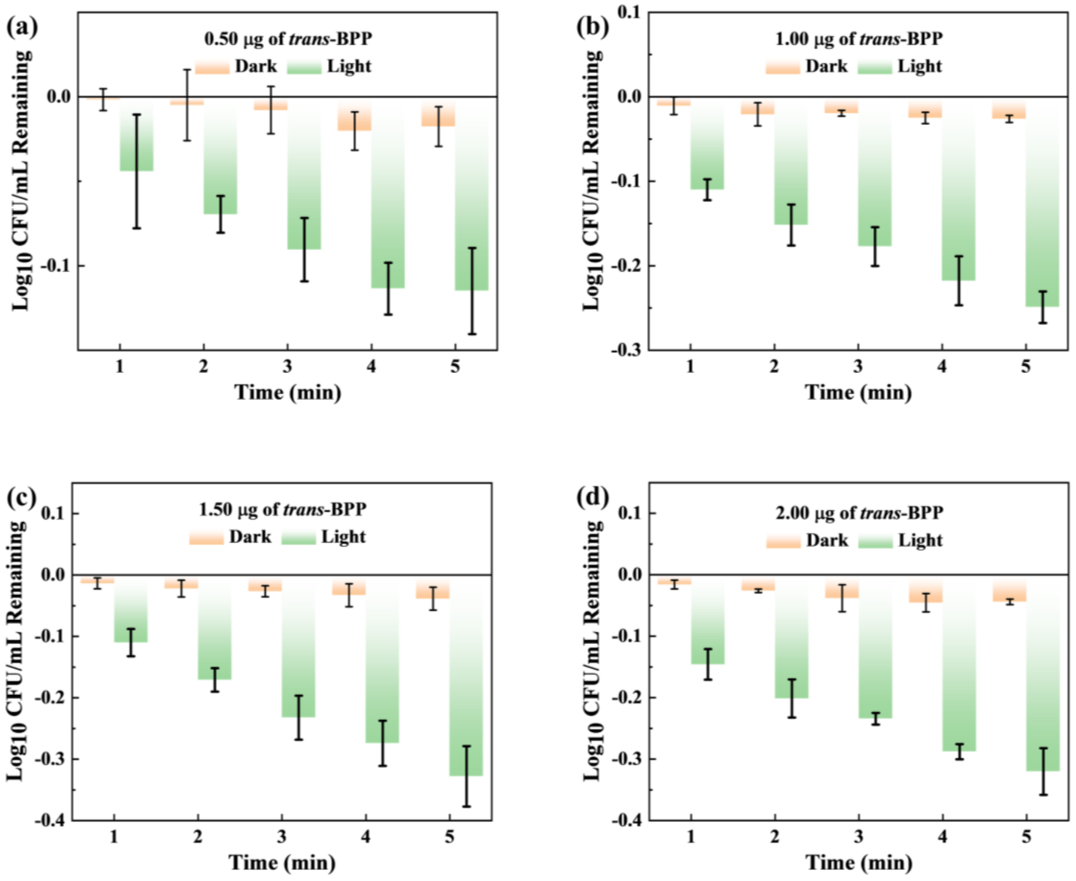

Figure S11. Anti-MDR *A. baumannii* performance of ***trans*-BPP** with concentration of 0.50 μg/mL (a), 1.00 μg/mL (b), 1.50 μg/mL (c) and 2.00 μg/mL (d) in dark and under white light irradiation for 1-5 min.

MRSA

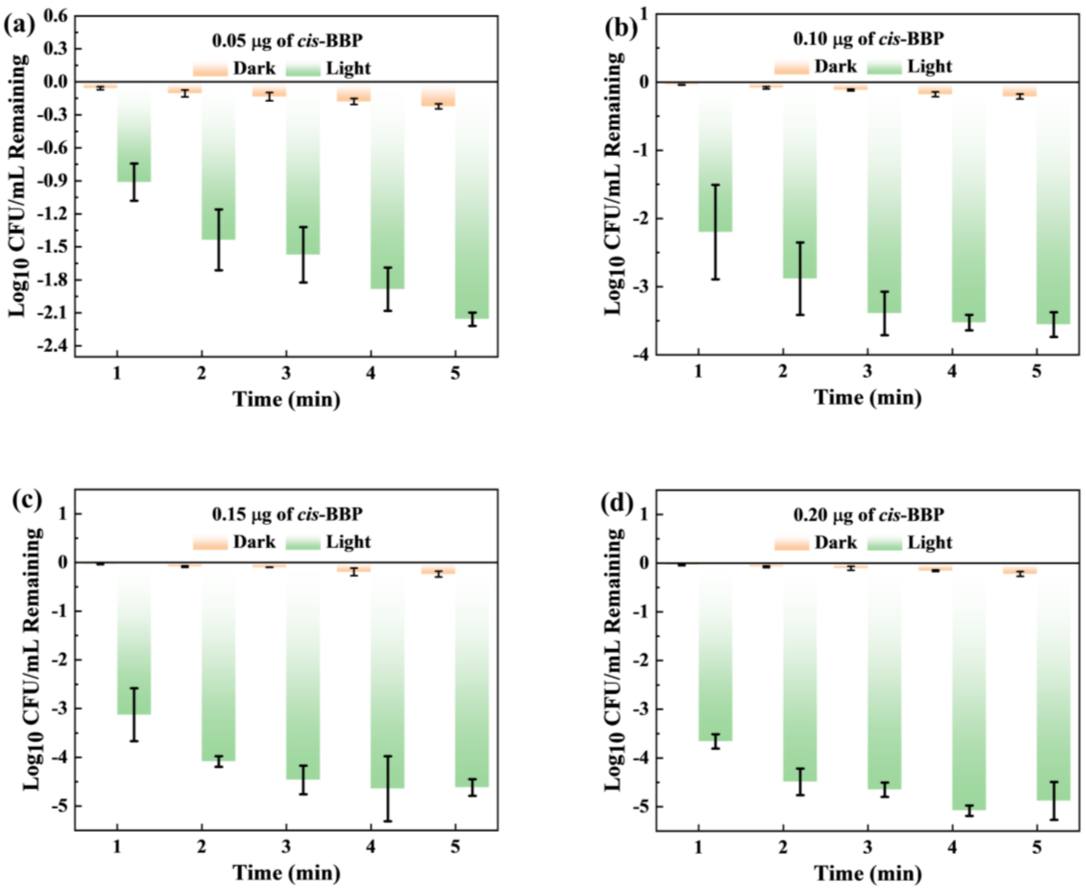

Figure S12. Anti-MRSA performance of ***cis*-BBP** with concentration of 0.05 μg/mL (a), 0.10 μg/mL (b), 0.15 μg/mL (c) and 0.20 μg/mL (d) in dark and under white light irradiation for 1-5 min.

MRSA

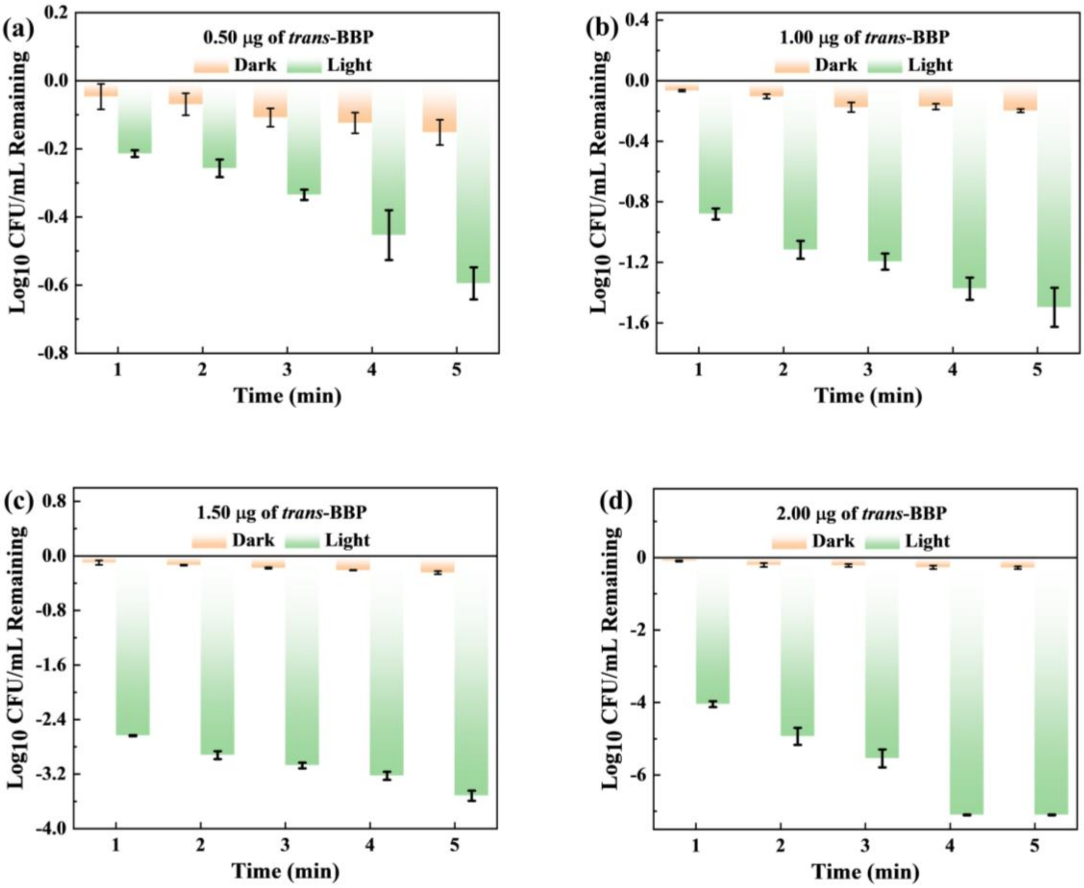

Figure S13. Anti-MRSA performance of ***trans*-BBP** with concentration of 0.50 μg/mL (a), 1.00 μg/mL (b), 1.50 μg/mL (c) and 2.00 μg/mL (d) in dark and under white light irradiation for 1-5 min.

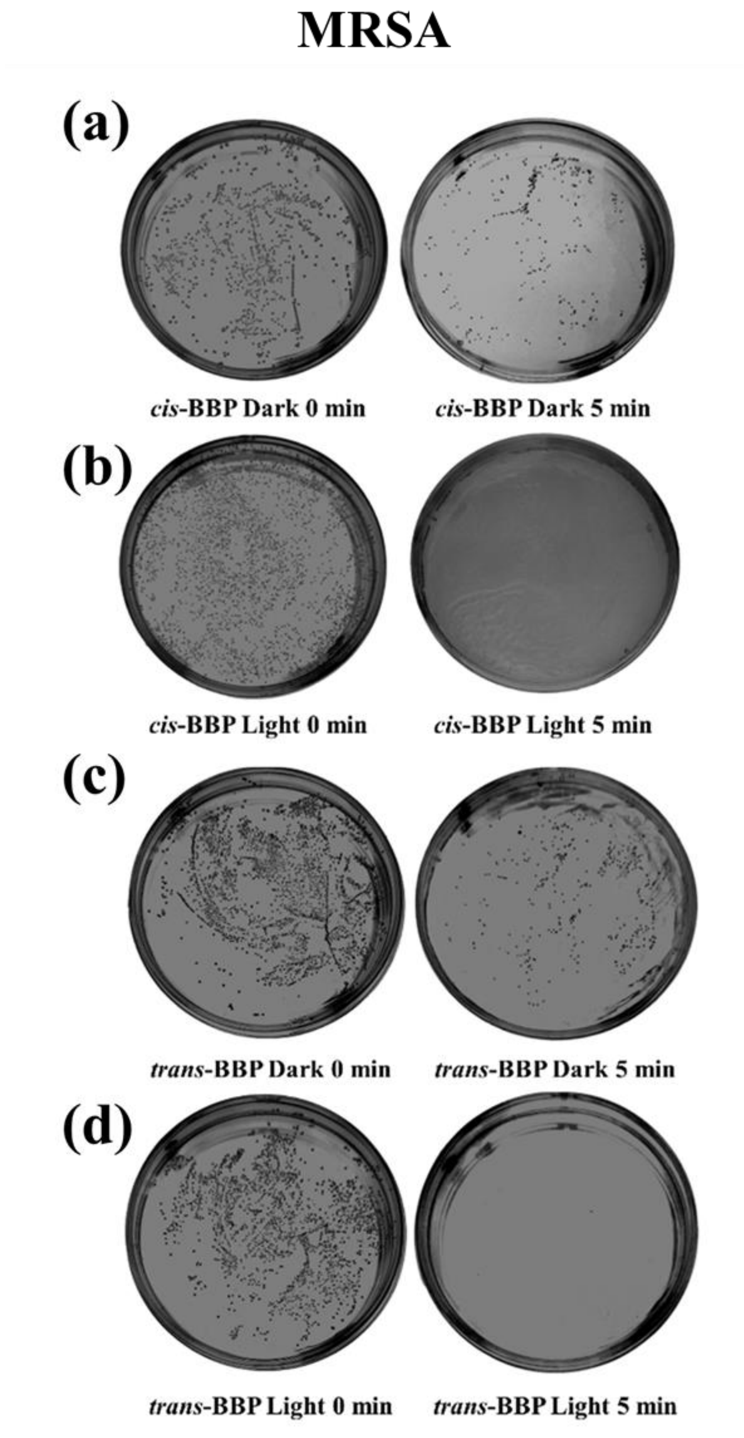

Figure S14. Photographs of MRSA colonies with exposure to ***cis*-BBP** (a) in dark and under light irradiation (b). And ***trans*-BBP** (c) in dark and under light irradiation (d).

MRSA

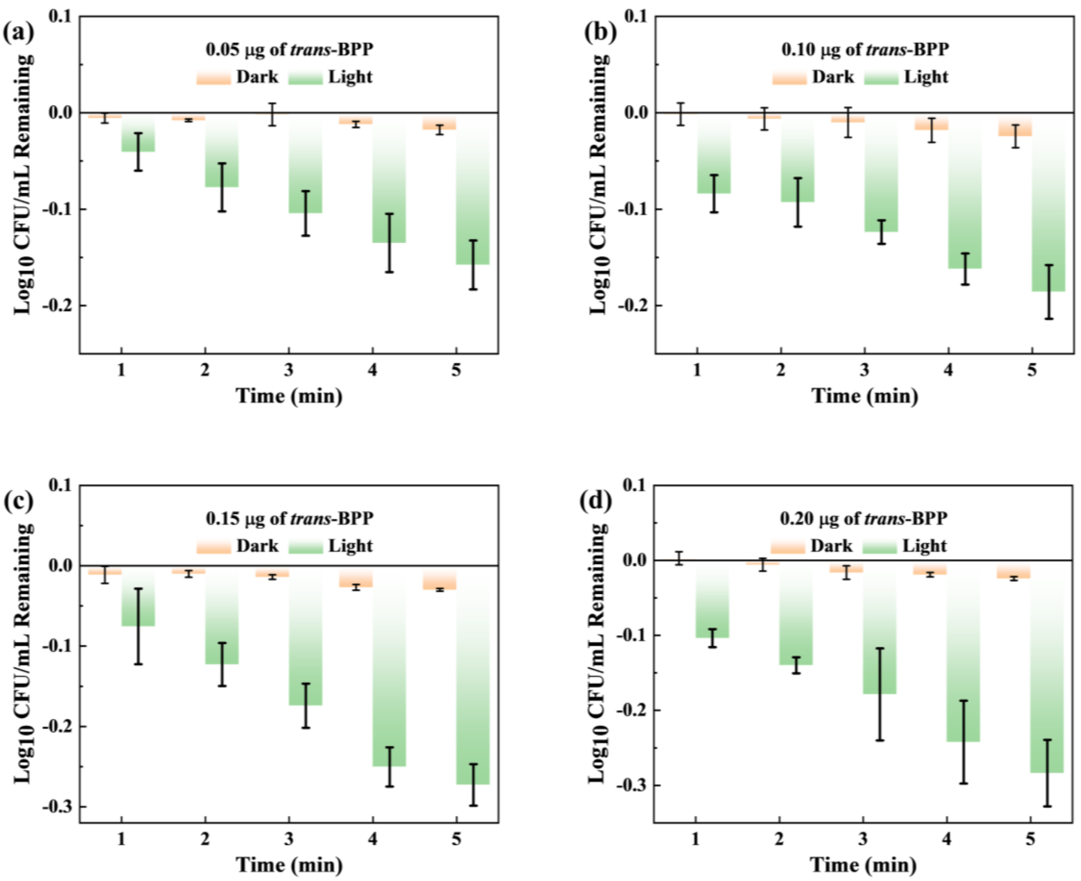

Figure S15. Anti-MRSA performance of ***cis*-BPP** with concentration of 0.05 μg/mL (a), 0.10 μg/mL (b), 0.15 μg/mL (c) and 0.20 μg/mL (d) in dark and under white light irradiation for 1-5 min.

MRSA

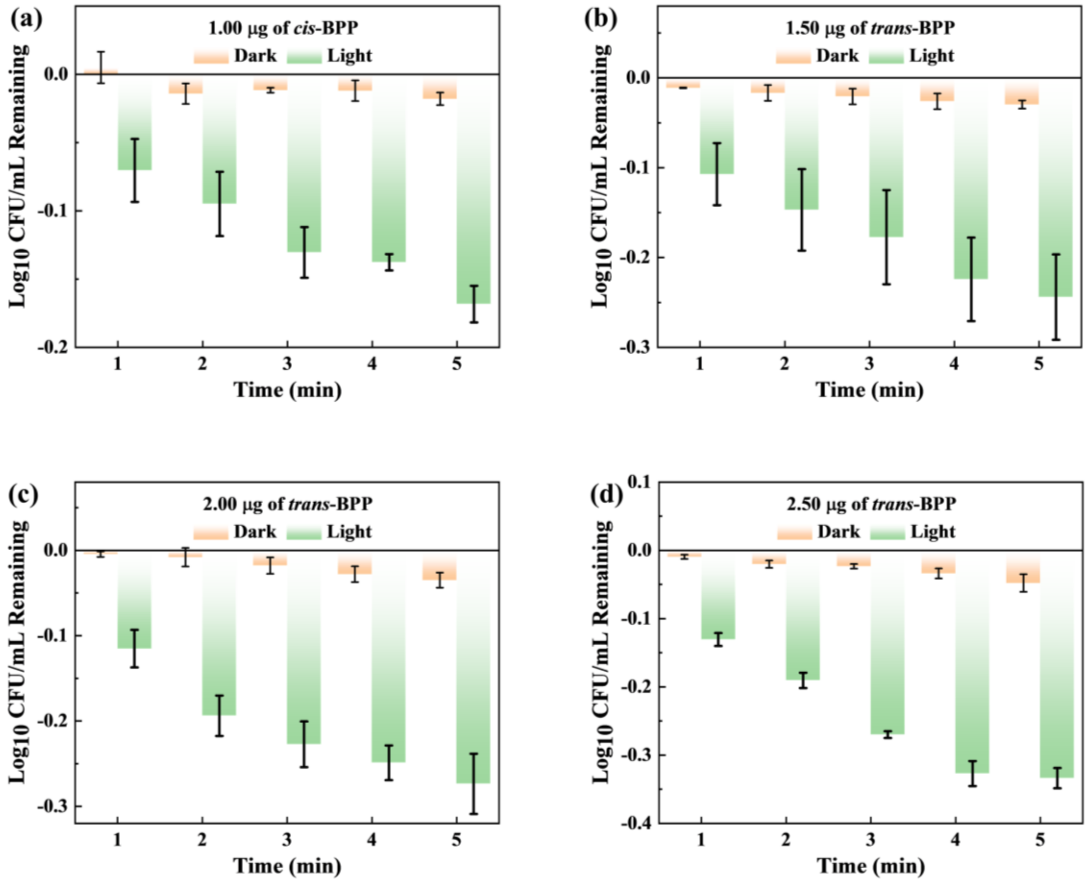

Figure S16. Anti-MRSA performance of ***trans*-BPP** with concentration of 0.50 μg/mL (a), 1.00 μg/mL (b), 1.50 μg/mL (c) and 2.00 μg/mL (d) in dark and under white light irradiation for 1-5 min.

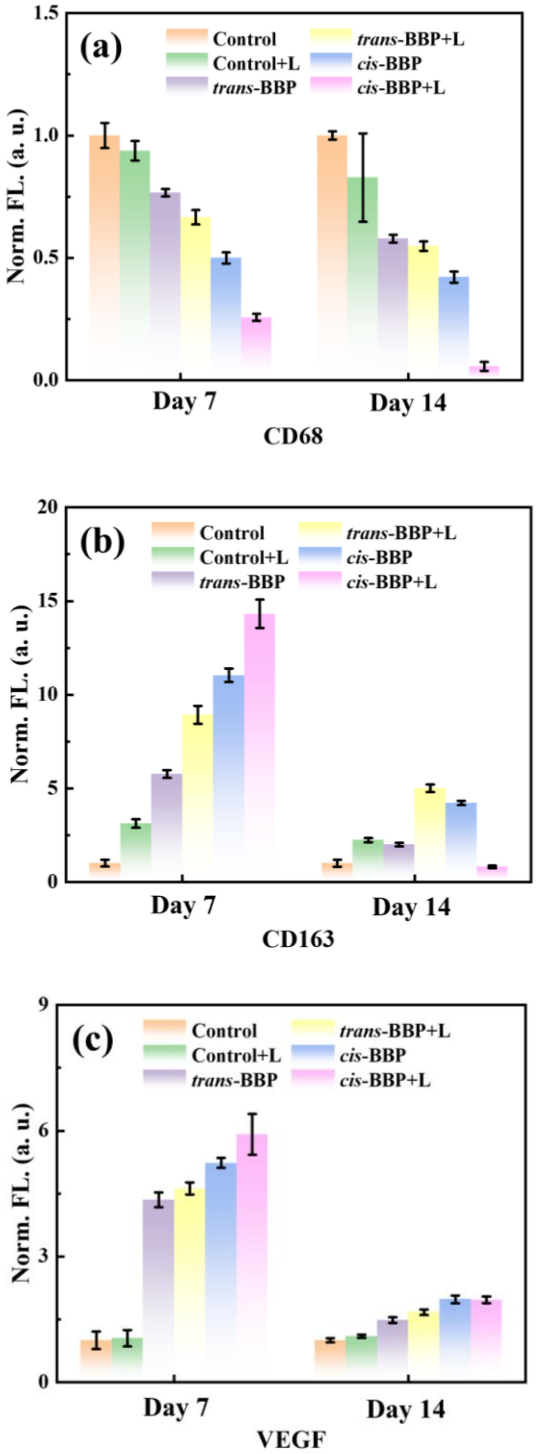

Figure S17. Fluorescent intensity ratio of CD68 (a), CD163 (b) and VEGF (c) against individual DAPI of infected mice wound with different treatments on day 7 and 14 post infection.

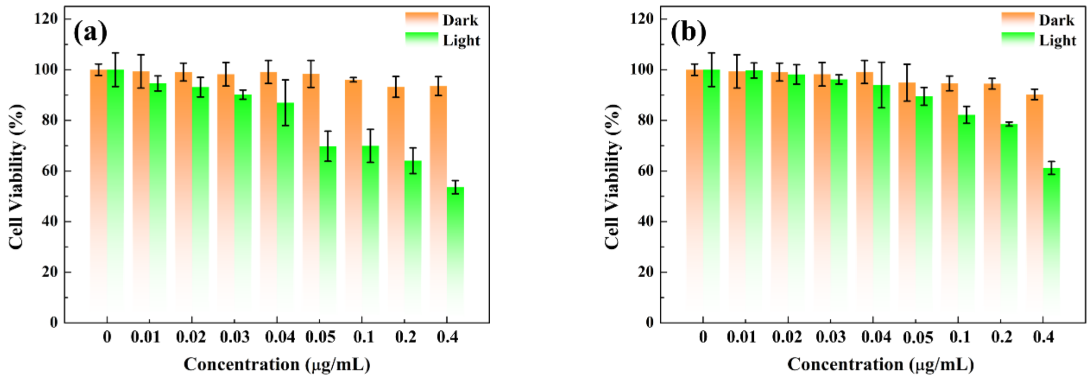

Figure S18. 24 Hours dark and photocytotoxicity of ***cis*-BBP** (a) and ***trans*-BBP** (b) on mouse fibroblast L929 cell line. Light irradiation dose was 30 J/cm^2.

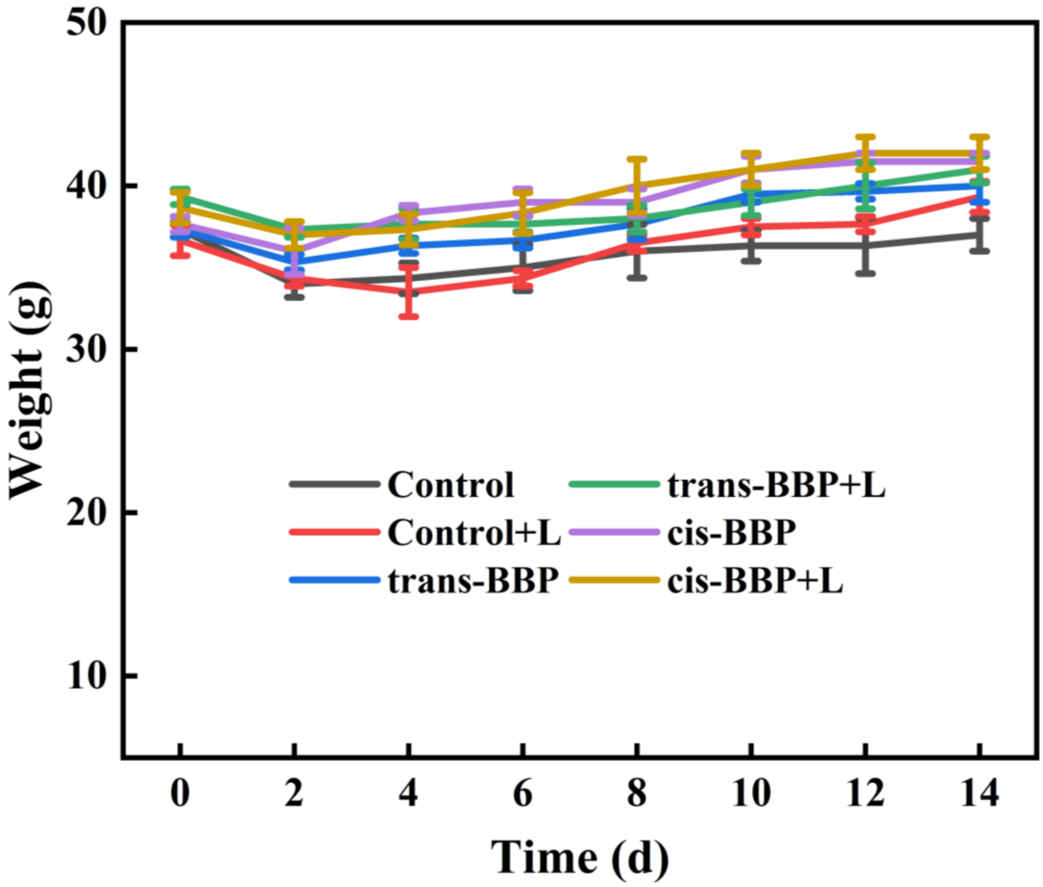

Figure S19. Time dependent MRSA infected ICR mice weight change with different treatments.

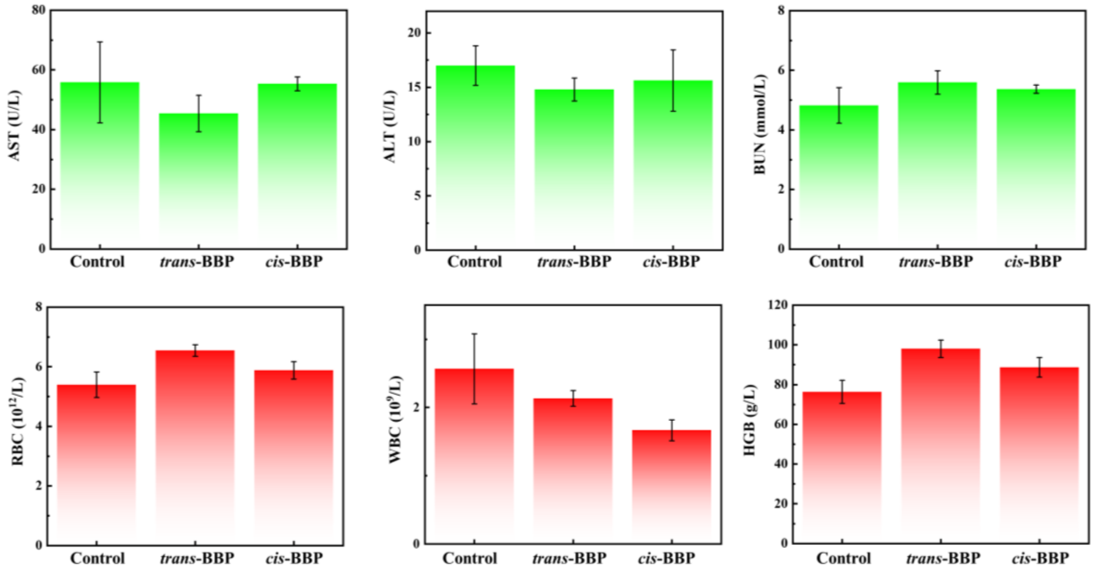

Figure S20. MRSA infected mice blood biochemistry and blood component count data collected at 14 days after injection. [Pt^{II}-complex] = 0.20 μg/mL, 1×PBS was used as control, injection volume was 200 μL.

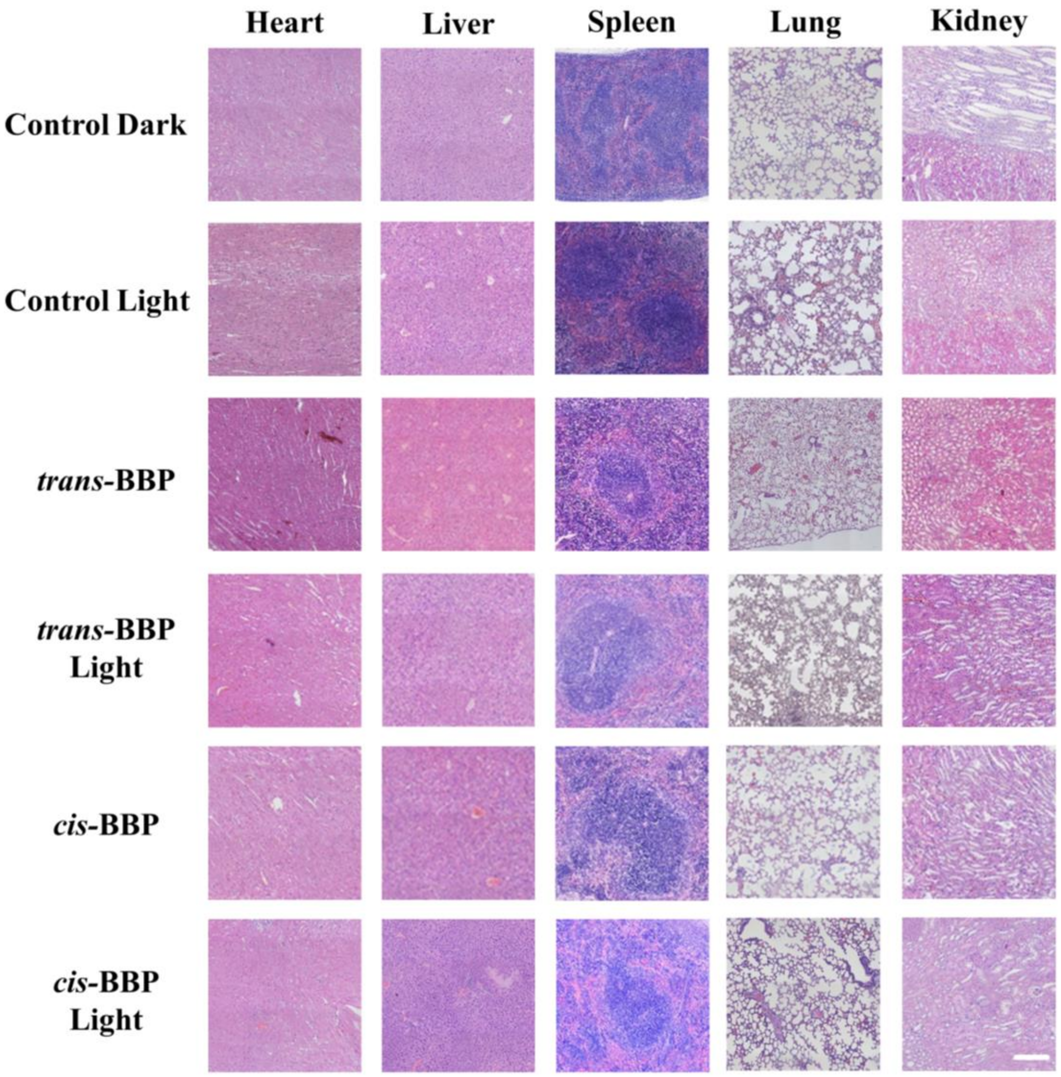

Figure S21. H&E staining of heart, liver, spleen, lung and kidney of infected ICR-mice harvested at 14 day with different treatments. Scale bar = 200 μm.

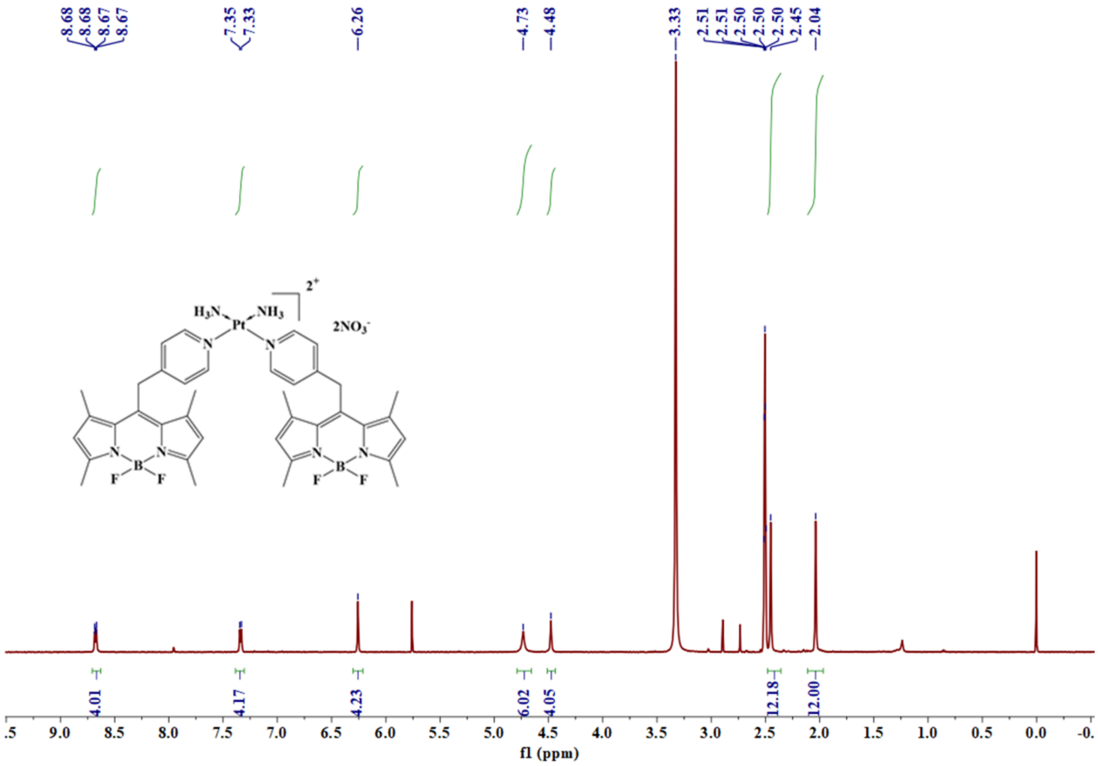

Figure S22. ^{1}H NMR of ***cis*-BBP** in DMSO-d_6, 300 MHz, 298 k.

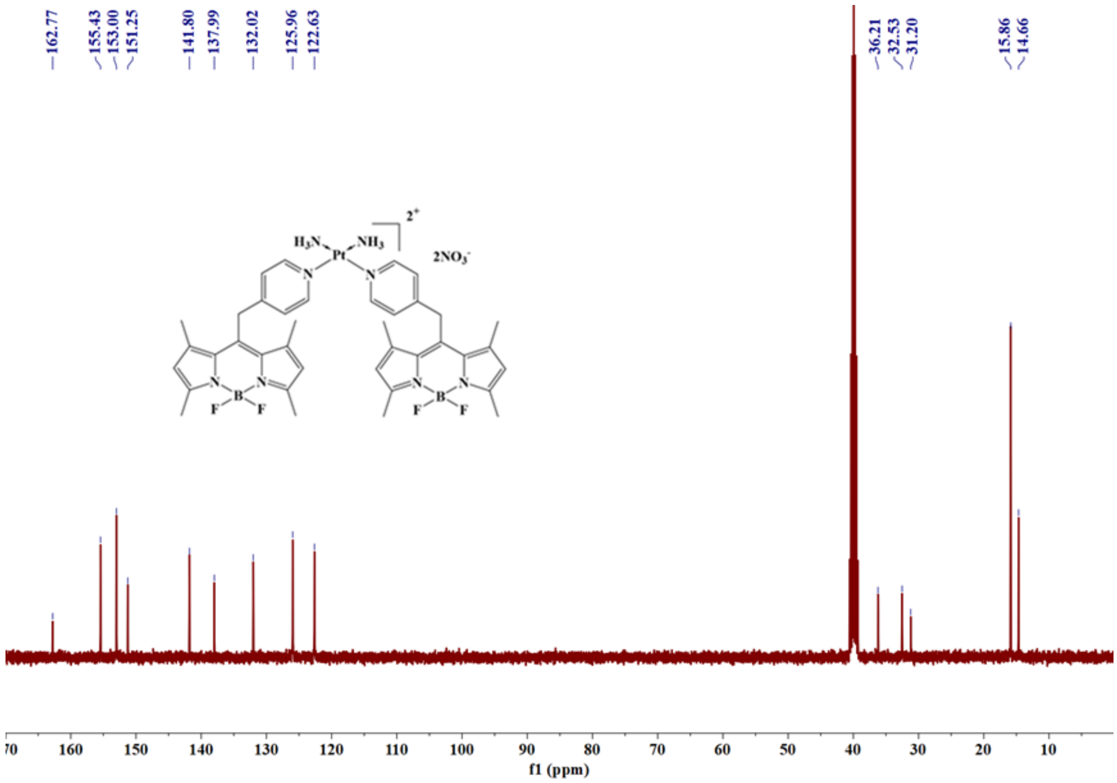

Figure S23. ^{13}C NMR of ***cis*-BBP** in DMSO-*d*₆, 150 MHz, 298 k.

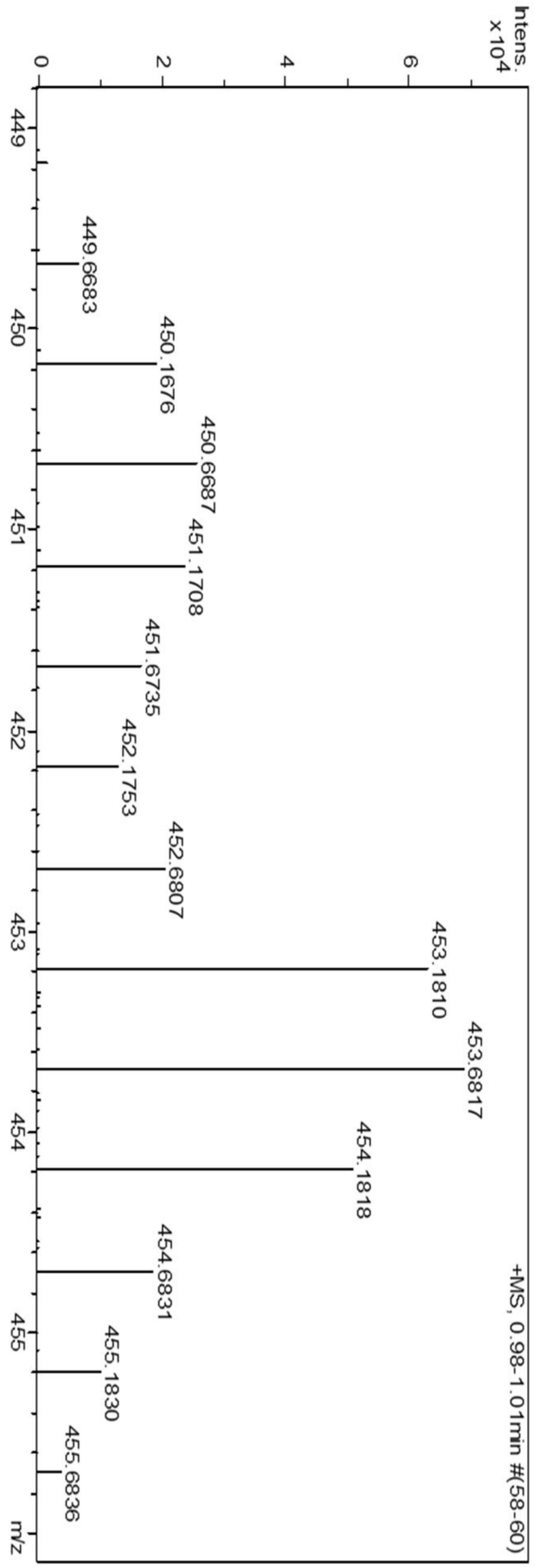

Figure S24. HRMS of ***cis*-BBP**.

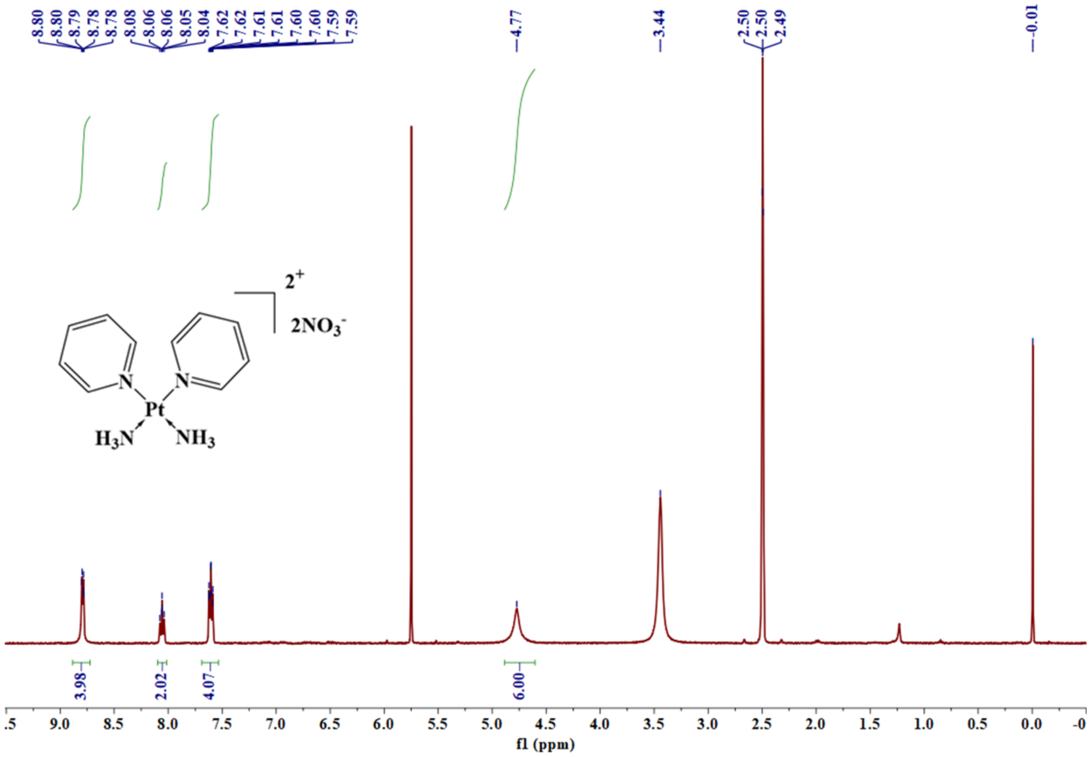

Figure S25. ^{1}H NMR of ***cis*-BPP** in DMSO-d_6, 300 MHz, 298 k.

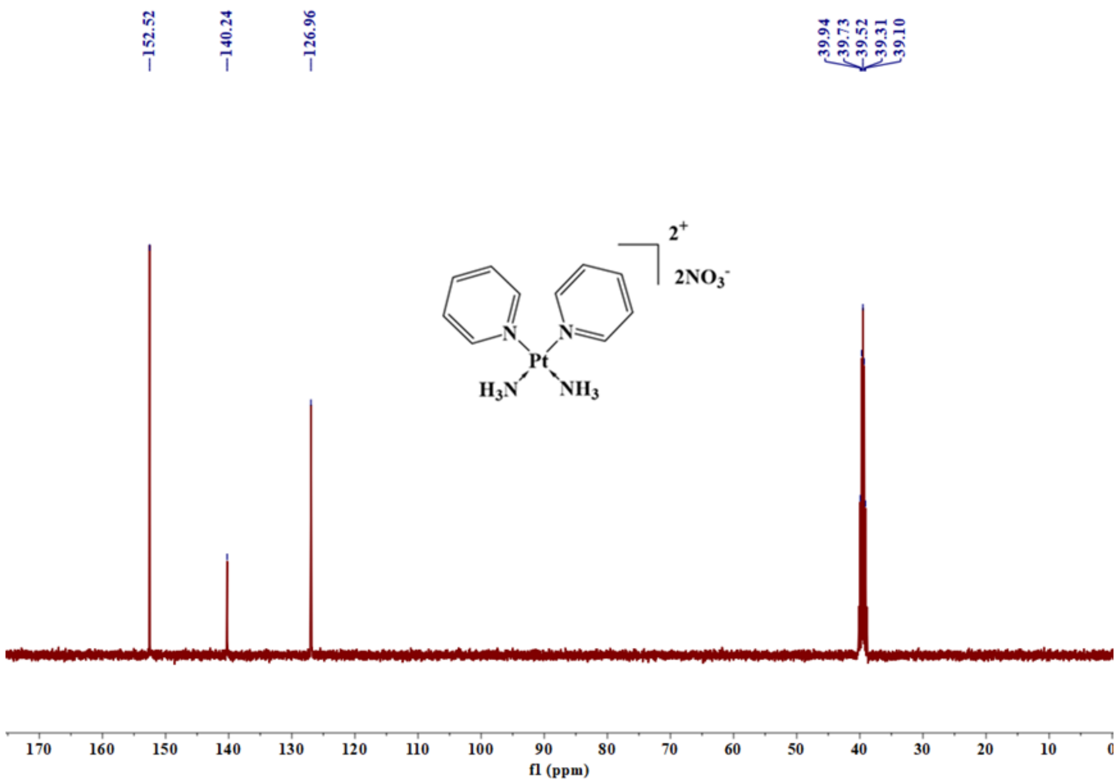

Figure S26. ^{13}C NMR of ***cis*-BPP** in DMSO-d_6, 150 MHz, 298 k.

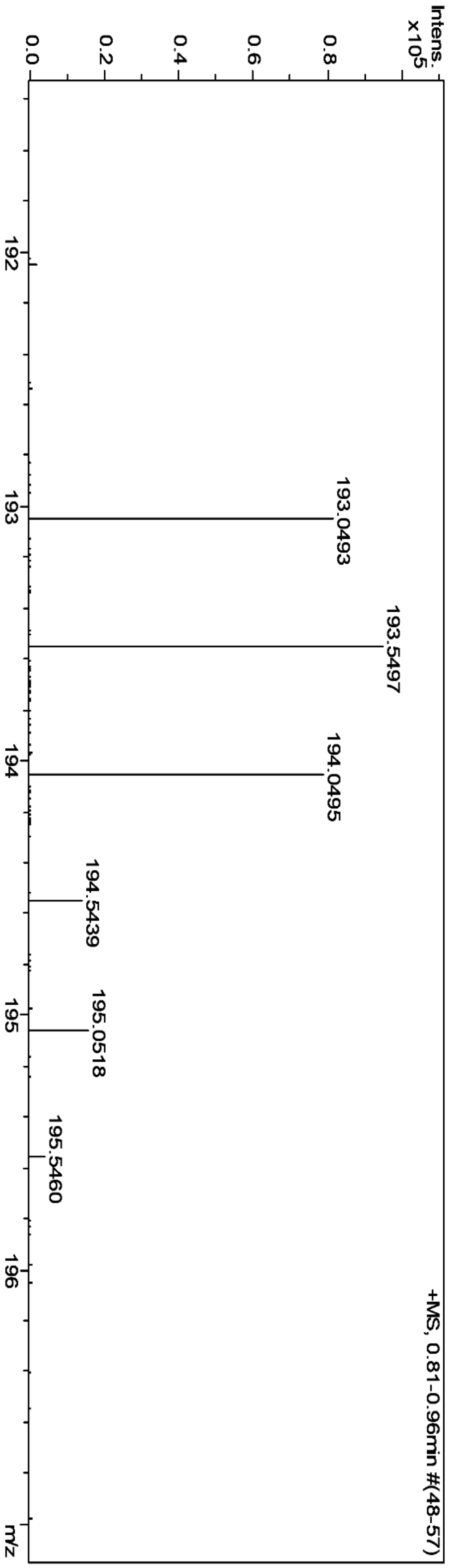

Figure S27. HRMS of ***cis*-BPP**.

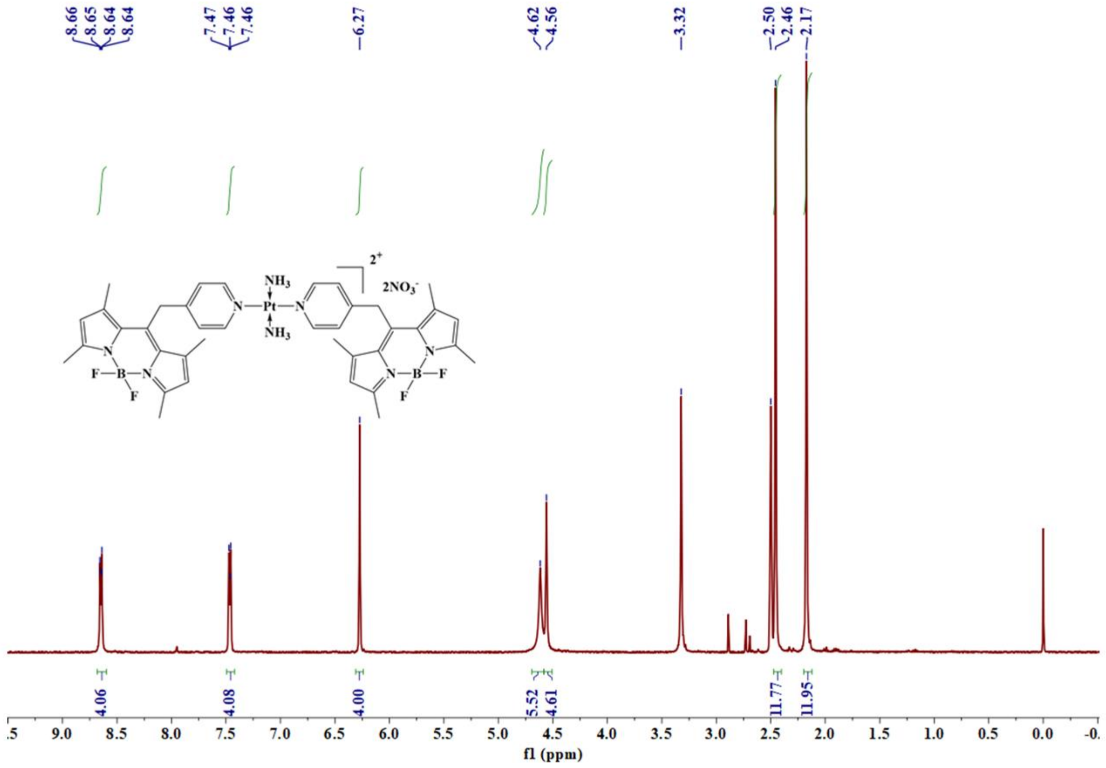

Figure S28. ^{1}H NMR of ***trans*-BBP** in DMSO-d_6, 300 MHz, 298 k.

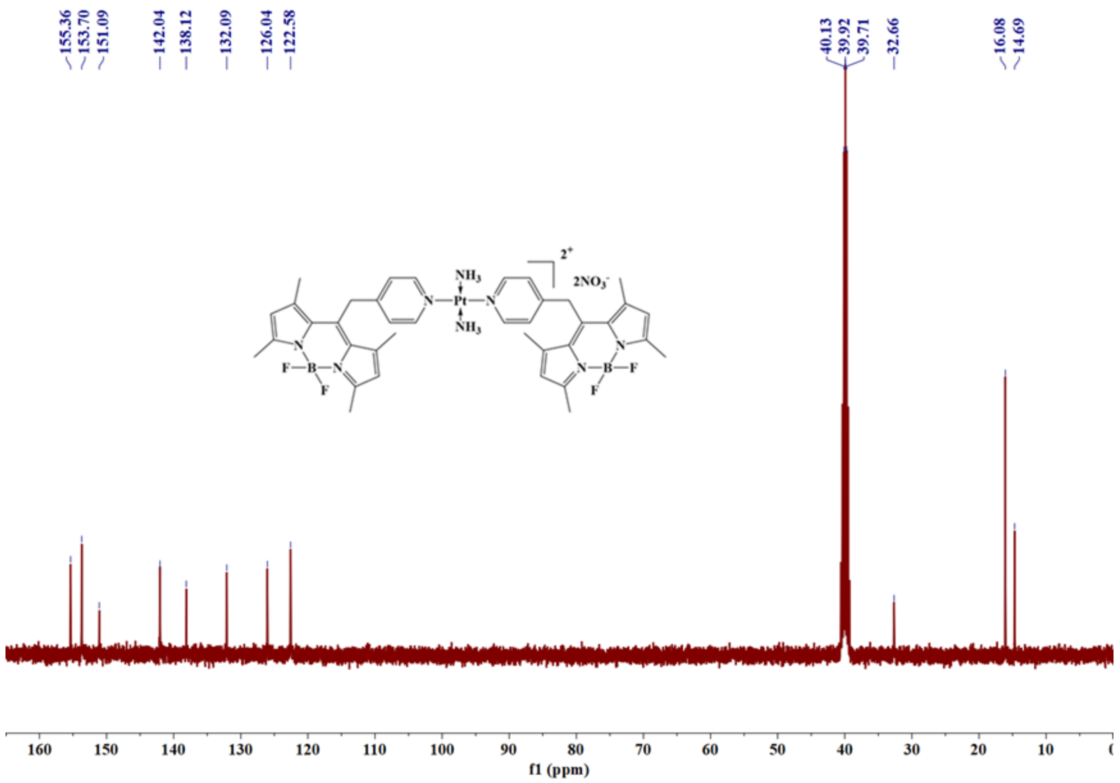

Figure S29. ^{13}C NMR of ***trans*-BBP** in DMSO-d_6, 150 MHz, 298 k.

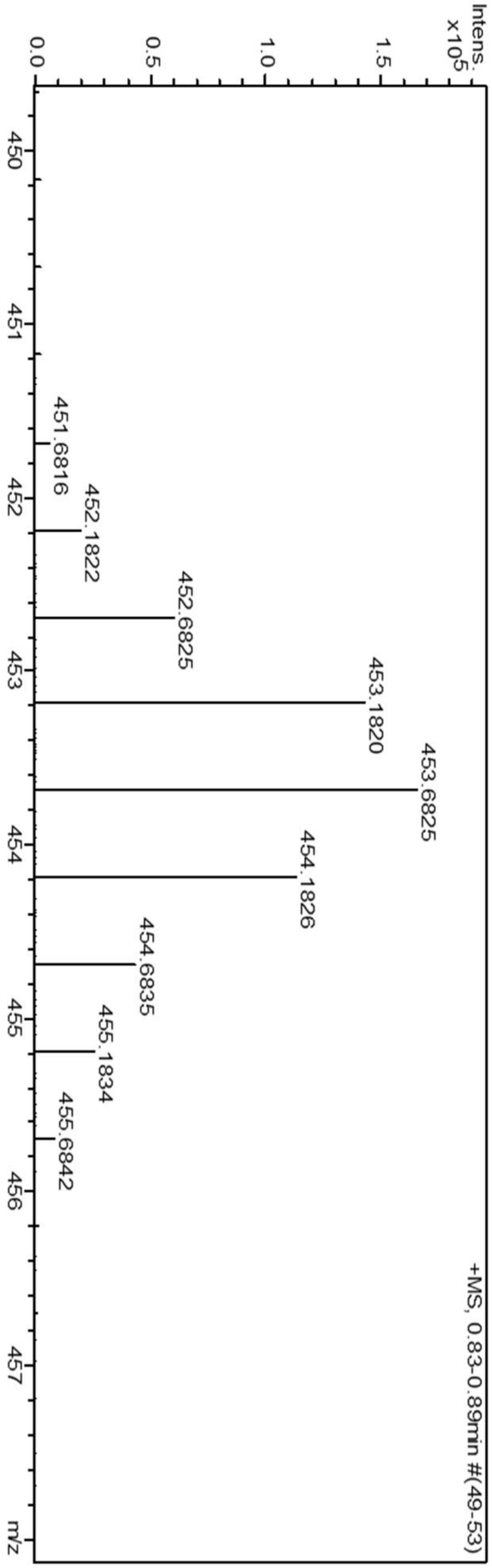

Figure S30. HRMS of ***trans*-BBP**.

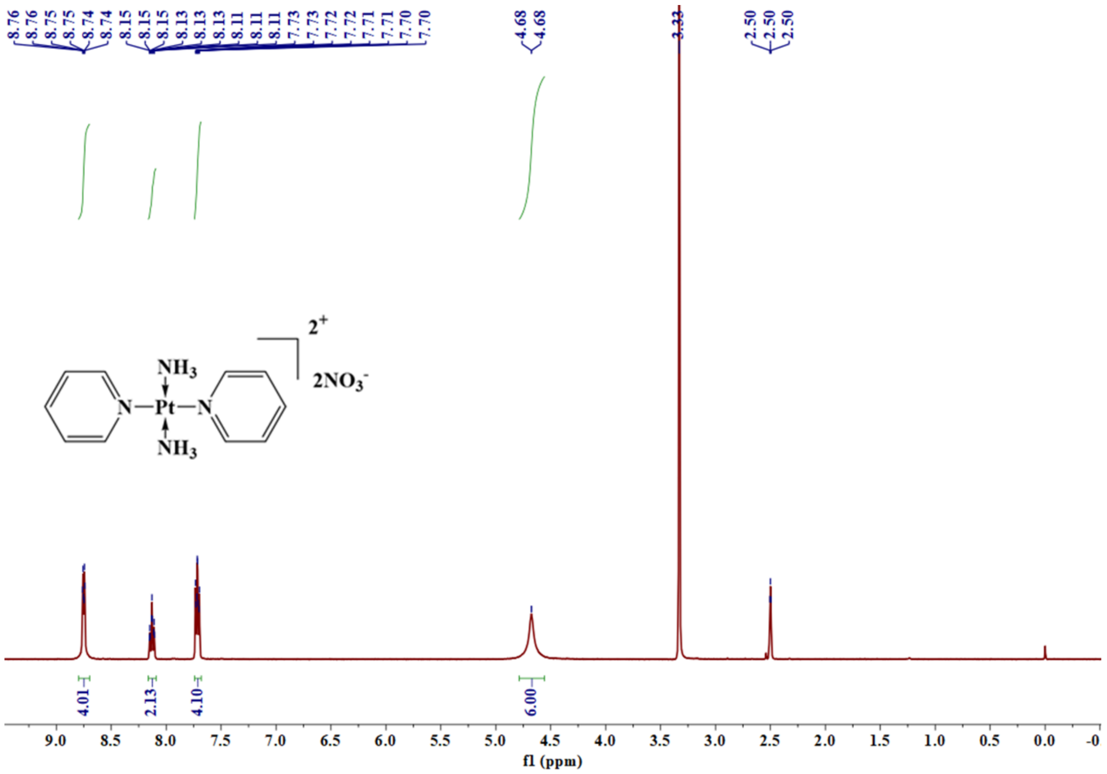

Figure S31. ^{1}H NMR of ***trans*-BPP** in DMSO-d_6, 300 MHz, 298 k.

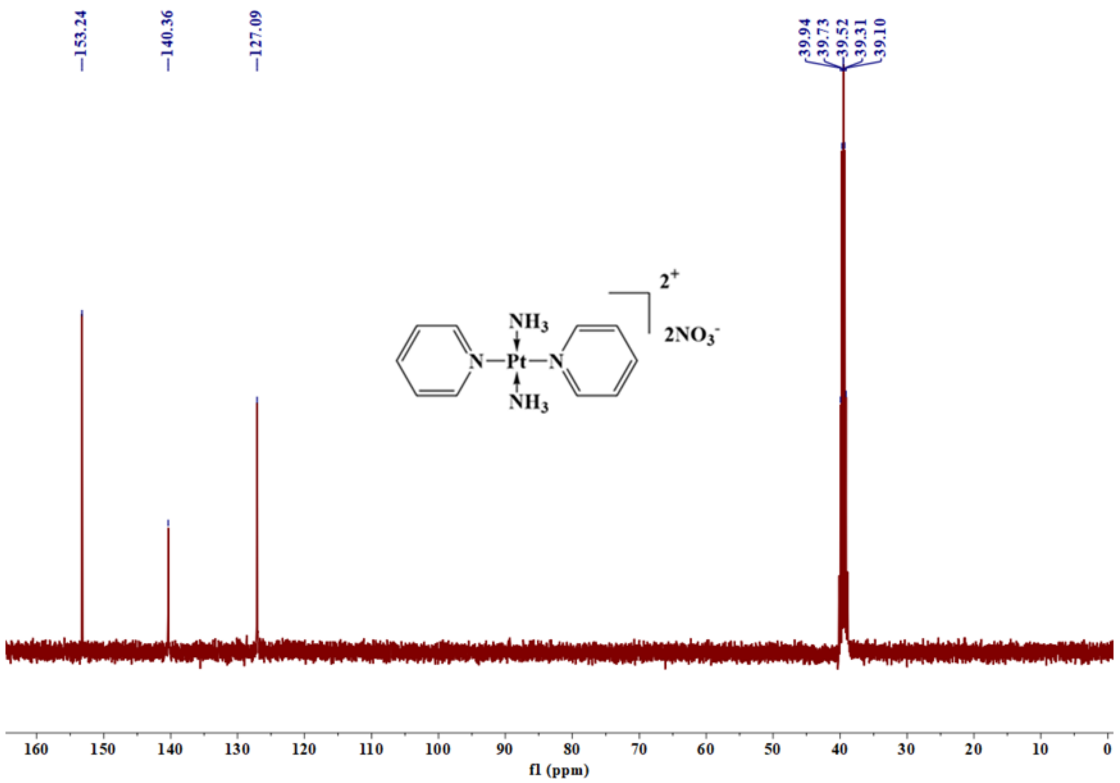

Figure S32. ^{13}C NMR of ***trans*-BPP** in DMSO-*d*$_6$, 150 MHz, 298 k.

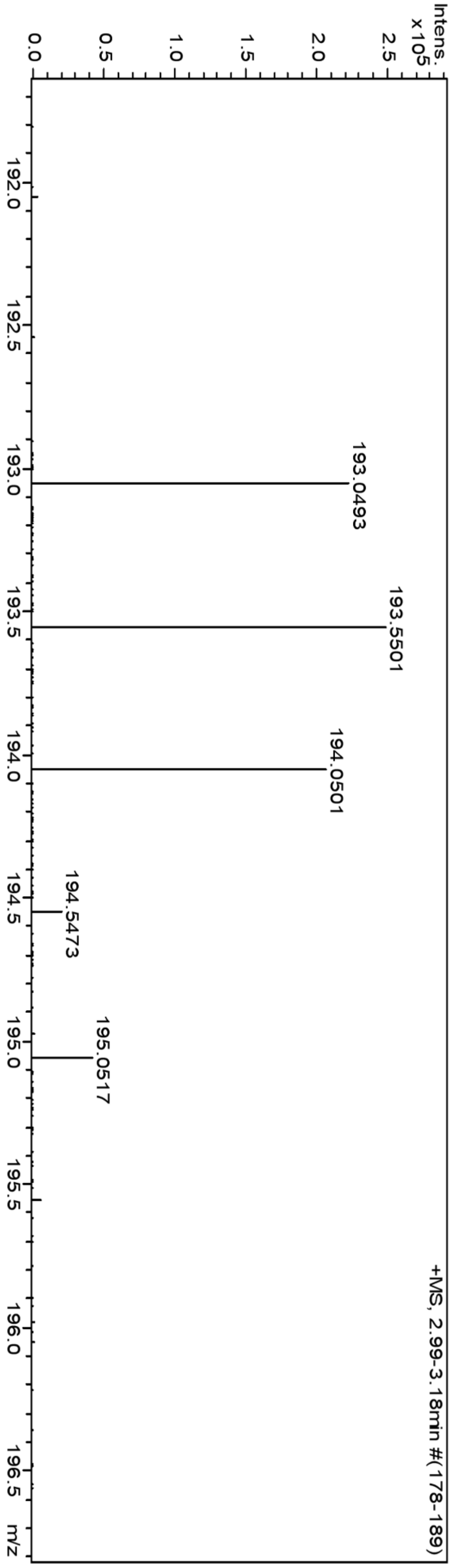

Figure S33. HRMS of ***trans*-BPP**.

Optimized geometries of *cis*-BBP and *trans*-BBP:

***cis*-BBP**:

C	1.17084400	1.67719700	-0.06142600
C	1.93982200	0.59593800	-0.46804200
C	2.45325600	0.55498300	-1.77579500
C	2.08181200	1.60145400	-2.63780500
C	1.28934500	2.64057200	-2.17618000
H	0.79604600	1.73617300	0.95986900
H	2.14887000	-0.19280500	0.25677500
H	2.41586400	1.61451600	-3.67734500
H	0.97746100	3.44644200	-2.84293700
Pt	-0.24674500	4.29503700	-0.23132000
N	0.86285000	2.70088700	-0.89195000
N	-1.47322900	5.88399200	0.43645600
H	-2.31558700	5.51237400	0.88947100
H	-1.02830500	6.50440900	1.12214700
H	-1.80752600	6.47712700	-0.33139300
N	1.19007200	5.62897300	-1.03891200
H	1.48567500	6.34890200	-0.37053600
H	2.04600100	5.12842700	-1.30223100
H	0.87065300	6.12107400	-1.88122300
N	-1.55073200	2.97261900	0.60483600
C	-1.58396600	2.82568400	1.95016800
C	-2.23774900	2.10108300	-0.17096000
C	-2.26309600	1.77726400	2.55136100
H	-1.01400600	3.54505500	2.54177700
C	-2.92725100	1.02753400	0.36960900
H	-2.19314700	2.25760300	-1.24939600
C	-2.92254000	0.82037400	1.76158700
H	-2.23682100	1.68407100	3.63873500
H	-3.41465500	0.33090300	-0.31587500
C	-3.55499600	-0.40294300	2.38771100
H	-2.95330400	-0.70857000	3.25462300
H	-4.53866700	-0.11925200	2.78238600
C	-4.91422400	-1.79743700	0.79161500
C	-6.27100000	-1.59455300	1.24537200
N	-4.97335800	-2.50309700	-0.41692100
C	-7.08300300	-2.19905400	0.29581700
C	-6.25929800	-2.72941200	-0.73454100
H	-8.16825500	-2.27487200	0.32785000
C	-3.67041100	-1.49165500	1.35146400
C	-2.47232300	-2.01292400	0.81284000
C	-1.11262400	-1.99791000	1.27489900

N	-2.50414500	-2.70626100	-0.39544800
C	-0.37264300	-2.69527800	0.31440300
C	-1.25621600	-3.10915600	-0.71146600
H	0.69119900	-2.91305800	0.36628900
B	-3.72784000	-2.57372500	-1.34531100
F	-3.80869500	-3.61686300	-2.23238500
F	-3.61194200	-1.32934600	-2.00388500
C	3.37003100	-0.54686400	-2.26517000
H	2.81725800	-1.13419500	-3.00853600
H	4.20490100	-0.08330500	-2.81067100
C	3.33913500	-2.71451100	-0.97922800
C	2.76814100	-3.65090600	-1.91406400
N	3.48202000	-3.37316300	0.24717200
C	2.60293200	-4.83863200	-1.20881300
C	3.02541400	-4.63427300	0.13089000
H	2.23117300	-5.78060400	-1.60770200
C	3.84508800	-1.41574600	-1.12953700
C	4.66955600	-0.83976200	-0.14299900
C	5.48639000	0.34149100	-0.13365600
N	4.82472600	-1.47419700	1.09034100
C	6.11330800	0.36285800	1.11186100
C	5.67213200	-0.75822200	1.85695300
H	6.84318900	1.09375200	1.45532200
B	3.82009700	-2.57118600	1.53339600
F	2.63335600	-1.90544300	1.94680400
F	4.32214200	-3.36990200	2.52476100
C	-0.93588400	-3.84983000	-1.96300300
H	0.10501800	-4.18828000	-1.93741400
H	-1.60086300	-4.71514100	-2.09032800
H	-1.08659400	-3.21251700	-2.85021400
C	-0.54509300	-1.46153800	2.55405600
H	-0.63500800	-0.36415500	2.62855600
H	-1.04660500	-1.88814100	3.43790500
H	0.52213400	-1.70693300	2.62251800
C	-6.69614200	-3.39922400	-1.98877800
H	-6.21891900	-4.38401800	-2.09603100
H	-7.78694700	-3.51773000	-1.99885600
H	-6.39539600	-2.80973200	-2.87011100
C	-6.76301800	-0.95879800	2.50980300
H	-6.27581300	-1.37914900	3.40369600
H	-6.60188200	0.13296600	2.51911800
H	-7.84386300	-1.12188800	2.61880500
C	2.96654000	-5.60333700	1.25815800
H	2.58619500	-6.57137900	0.90853800

H	3.95896700	-5.74260500	1.71113100
H	2.30768000	-5.23205200	2.05948900
C	2.47576300	-3.47475700	-3.37283800
H	1.63707800	-2.78005100	-3.54903500
H	3.34936100	-3.10030100	-3.92914800
H	2.19170900	-4.43677800	-3.82059400
C	6.03096100	-1.12536800	3.25443800
H	6.45856400	-2.13794000	3.29830900
H	6.75648300	-0.40992600	3.66222700
H	5.13973600	-1.13675500	3.90210600
C	5.73377100	1.32797000	-1.23384800
H	6.11835800	0.84219500	-2.14538200
H	4.82423000	1.88474300	-1.51696200
H	6.48541900	2.06489000	-0.91896400

***trans*-BBP**:

C	-2.97699100	-0.58440800	-0.08643800
C	-4.27860100	-0.52784100	0.38771100
C	-4.52050800	-0.57374000	1.77404700
C	-3.39990500	-0.67914400	2.61624800
C	-2.12119400	-0.72509800	2.07795400
H	-2.76867000	-0.54632300	-1.15746100
H	-5.08932400	-0.42386000	-0.33716000
H	-3.51782300	-0.71544100	3.70118200
H	-1.23698300	-0.79394400	2.71535800
Pt	0.00075800	-0.69225600	-0.02920100
N	-1.90878500	-0.67605500	0.74233900
N	0.00397700	1.39772300	-0.04369700
H	0.94606200	1.78628600	0.07359100
H	-0.36096500	1.76930400	-0.92848400
H	-0.57850100	1.79183800	0.70270400
N	-0.00348000	-2.78180300	-0.02081900
H	-0.29868400	-3.16266300	-0.92734600
H	0.92555900	-3.16989300	0.17474200
H	-0.64378700	-3.16724800	0.68131200
N	1.91672700	-0.69154500	-0.78551200
C	2.15011900	-0.75920400	-2.11630500
C	2.97203000	-0.58695600	0.05876100
C	3.43701300	-0.71966600	-2.63501900
H	1.27642700	-0.83760800	-2.76655600
C	4.28099500	-0.53577800	-0.39557700
H	2.74664800	-0.53412700	1.12599700
C	4.54433400	-0.60094400	-1.77720100
H	3.57190700	-0.77118500	-3.71735300
H	5.08018700	-0.42073700	0.34032300

C	5.95326000	-0.54889800	-2.32874300
H	6.22517400	-1.56338300	-2.64664600
H	5.95595500	0.06288500	-3.24009700
C	6.81534700	1.33559900	-0.93228500
C	6.26995400	2.47735900	-1.61338100
N	7.37319700	1.78127000	0.26602400
C	6.53815900	3.57378400	-0.79429900
C	7.20307900	3.11426500	0.36920500
H	6.30707300	4.61485600	-1.01294800
C	6.90052700	-0.02783300	-1.28011100
C	7.67392800	-0.92424200	-0.53182100
C	8.11123100	-2.27927700	-0.76975200
N	8.24914900	-0.49958900	0.67171600
C	8.94133900	-2.60321900	0.29591400
C	8.99073300	-1.49611000	1.18526000
H	9.48339200	-3.53725700	0.43151300
B	7.70466600	0.76572800	1.39550300
F	8.60354100	1.27117500	2.29678500
F	6.48225000	0.40626800	2.00870300
C	-5.92082900	-0.51571500	2.34652200
H	-6.18620300	-1.52595100	2.68295400
H	-5.91033900	0.10890900	3.24907500
C	-7.66846600	-0.91878900	0.58181600
C	-8.09928100	-2.27116800	0.84512900
N	-8.26314200	-0.51182000	-0.61839000
C	-8.94518200	-2.61129600	-0.20294400
C	-9.01054800	-1.51669200	-1.10659800
H	-9.48735600	-3.54810900	-0.31724400
C	-6.88514600	-0.01081500	1.30553700
C	-6.80780400	1.34773200	0.93758300
C	-6.25407200	2.49977200	1.59420300
N	-7.38485200	1.77575900	-0.25805900
C	-6.53683900	3.58421200	0.76422000
C	-7.21879000	3.10743700	-0.38237300
H	-6.30404600	4.62857000	0.96460100
B	-7.73204700	0.74410600	-1.36803500
F	-6.51885700	0.37785300	-1.99519700
F	-8.64566300	1.23578800	-2.26211600
C	-7.81198700	-3.14813300	2.02621000
H	-6.75510900	-3.46269100	2.06920000
H	-8.05489300	-2.65347200	2.98018400
H	-8.41608600	-4.06407900	1.97187200
C	-9.74063100	-1.45080000	-2.40124200
H	-10.31634600	-2.37097800	-2.56244400

H	-10.41914900	-0.58572400	-2.42678100
H	-9.03837900	-1.31929000	-3.24023100
C	-7.67808300	3.88827900	-1.56396800
H	-8.75787600	3.75647600	-1.72691400
H	-7.46197000	4.95521200	-1.42362500
H	-7.18189000	3.54198600	-2.48471500
C	-5.58327300	2.60083500	2.93082000
H	-6.22311100	2.22459400	3.74563400
H	-4.63254100	2.04162600	2.97275200
H	-5.35440000	3.65073700	3.16016200
C	7.84400200	-3.17291700	-1.94301400
H	6.78848500	-3.49011200	-1.99824800
H	8.10093100	-2.69085200	-2.89975600
H	8.44879200	-4.08683200	-1.86667600
C	5.62001900	2.55857400	-2.96162700
H	6.27302200	2.17166800	-3.76084400
H	4.67063600	1.99768600	-3.01049000
H	5.39364300	3.60481700	-3.20941900
C	7.64297800	3.91237200	1.54665900
H	8.72015200	3.78401900	1.72854400
H	7.42825300	4.97696700	1.38765800
H	7.13253800	3.57883900	2.46434000
C	9.70056900	-1.41078700	2.48997100
H	10.27594400	-2.32717300	2.67255000
H	10.37644800	-0.54362000	2.51411900
H	8.98512100	-1.26972900	3.31616500
